# Dravet syndrome: novel insights into *SCN1A*-mediated epileptic neurodevelopmental disorders within the molecular diagnostic-therapeutic framework

**DOI:** 10.3389/fnins.2025.1634718

**Published:** 2025-07-23

**Authors:** Guirui Zhang, Shupeng Huang, Mingzhen Wei, Yongmo Wu, Zhengyi Xie, Jin Wang

**Affiliations:** ^1^Department of Medical Oncology, Liuzhou Workers' Hospital, Liuzhou, China; ^2^Department of Medicine, Guangxi University of Science and Technology, Liuzhou, China; ^3^Guangxi Key Laboratory of Parkinson’s Disease Diagnosis and Treatment, The First Affiliated Hospital of Guangxi University of Science and Technology, Guangxi Zhuang Autonomous Region, Liuzhou, China

**Keywords:** Dravet syndrom, SCN1A, GABAergic, antisence oligonucleotides, molecular

## Abstract

Dravet Syndrome (DS), a rare genetic encephalopathy characterized by severe drug-resistant epilepsy and progressive neurodevelopmental regression in infancy, is caused by *de novo* mutations in the *SCN1A* gene on chromosome 2q24 in over 80% of cases. This review synthesizes current insights into its molecular pathogenesis, precision diagnostics, and therapeutic innovations: *SCN1A* mutations disrupt Nav1.1 sodium channel expression and membrane trafficking in GABAergic interneurons through transcriptional dysregulation, pre-mRNA splicing defects, and gating dysfunction, thereby impairing inhibitory synaptic transmission and disrupting brainwide excitatory-inhibitory balance. Notably, polygenic interactions (e.g., *DEPDC5*, *CHD2* variants), astrocytic calcium signaling aberrations, and mitochondrial metabolic deficits synergistically exacerbate network hyperexcitability. Diagnostic advancements include a stratified framework integrating early febrile seizure phenotypes, comprehensive *SCN1A* sequencing (including deep intronic variants), and multimodal assessments (e.g., *γ*-band EEG power analysis and hippocampal volumetry), which significantly accelerate clinical diagnosis and reduce misdiagnosis. Therapeutic strategies are evolving from empirical seizure control to mechanism-targeted interventions: antisense oligonucleotides (ASOs) restore *SCN1A* transcript integrity by blocking pathogenic exon inclusion; adeno-associated virus (AAV9)-mediated activation of GABAergic neuron-specific *SCN1A* promoters and CRISPR/dCas9-driven endogenous Nav1.1 upregulation have both been shown to improve inhibitory synaptic function and elevate seizure thresholds in preclinical models. Additionally, novel molecules such as the Nav1.1-selective agonist Hm1a and 5HT_2_BR receptor modulators offer new avenues by remodeling neuronal electrophysiology and neurotransmitter homeostasis. By dissecting the multi-dimensional molecular networks underlying DS and highlighting interdisciplinary integration of diagnostic-therapeutic technologies, this review provides a theoretical foundation for developing *SCN1A*-centric precision medicine, advocating a shift from symptomatic management to mechanism-driven interventions in clinical practice.

## 1 Introduction

DS, first described as severe myoclonic epilepsy of infancy (SMEI) by Charlotte Dravet in 1978 and renamed in 1989, is a rare early-onset genetic epileptic encephalopathy. Molecular characterization in 2001 identified *de novo* mutations in the *SCN1A* gene on chromosome 2q24 as the underlying cause for over 80% of cases (Claes et al., 2001). *SCN1A* encodes the α-subunit of the voltage-gated sodium channel Nav1.1, which is essential for maintaining excitability in GABAergic interneurons by mediating the sodium influx that drives action potential upstroke; normally, Nav1.1 initiates action potentials via rapid sodium influx and terminates depolarization through fast inactivation to ensure precise neuronal excitability (Chilcott et al., 2022). Reduced Nav1.1 function slows depolarization, decreases action potential amplitude, and directly impairs inhibitory neuronal firing, while its inactivation or reduced expression prolongs action potential depolarization, lowers the threshold for repetitive firing, triggers neuronal hyperexcitability, and leads to epilepsy (e.g., Dravet syndrome) and neurodevelopmental deficits (Black and Waxman, 2013).

Approximately 80% of *SCN1A* missense mutations selectively compromise voltage-gating properties of Nav1.1 in GABAergic neurons, disrupting the balance between inhibitory GABAergic and excitatory glutamatergic transmission. This attenuation of inhibitory input not only disinhibits pyramidal neurons but also triggers cascading effects in dopaminergic and serotonergic systems, amplifying abnormal network synchronization and forming the electrophysiological basis for seizures and progressive neurodevelopmental deficits (Chilcott et al., 2022; Li et al., 2023).

The clinical course is characterized by a distinct trajectory: febrile seizures in the first year of life evolve into multiple seizure types—myoclonic, absence, focal—accompanied by progressive motor-cognitive decline, culminating in permanent ataxia, language/visual-perceptual deficits, and psychiatric symptoms in adulthood. The high risk of status epilepticus and SUDEP establishes DS as one of the most lethal epileptic encephalopathies in childhood (Anwar et al., 2019).

Epidemiological studies report a global incidence of 1/15,000 to 1/40,000 without sex bias, with a median diagnostic delay of 1.6–9.2 years. During this period, nearly 70% of patients experience at least one episode of status epilepticus, significantly increasing the risk of neuronal injury and SUDEP (Anwar et al., 2019).

Burden-of-disease analyses reveal profound societal impacts: a 3.7–20.8% mortality risk dominated by SUDEP, a 38% reduction in quality of life (Kiddy KINDL score), and a caregiving burden characterized by 70% caregiver depression, annual healthcare costs exceeding $77,000, and maternal productivity losses of $17,600/year—11 times higher than paternal costs (Strzelczyk et al., 2023).

Despite its low global incidence of 2.2–6.5 cases per 100,000 population, DS imposes significant challenges due to progressive neurological decline, alarmingly high mortality (15.84 deaths per 1,000 person-years, primarily from SUDEP), and delayed diagnosis (median age 1.6–9.2 years; Sullivan et al., 2024). These unmet needs underscore the critical importance of developing *SCN1A*-targeted precision medicine approaches, which this review aims to systematically address.

## 2 New interpretation of mechanisms

### 2.1 Dysregulation of *SCN1A* transcription and splicing: bidirectional pathogenesis driven by non-coding variants

The positional specificity of *SCN1A* intronic variants dictates splicing patterns and phenotypic gradients: canonical splice site variants such as c.602 + 1G > A induce complete exon skipping, leading to near-abolition of full-length mRNA and haploinsufficiency-mediated severe DS, whereas deep intronic variants such as c.4853-25 T > A cause partial exon skipping or intron retention, maintaining higher full-length mRNA levels and correlating with milder febrile epilepsy. The significant negative correlation between full-length mRNA retention, variant location, and phenotypic severity (Spearman r = −0.643, *p* < 0.001) provides robust evidence for splicing dysregulation as the core driver of phenotypic diversity (Zhou et al., 2021).

Developmental stage-specific splicing anomalies offer a dynamic pathogenic perspective: in a knock-in mouse model of intron 20 variant c.3969 + 2451G > C, aberrant inclusion of poison exon 20 N reduces *SCN1A* mRNA and Nav1.1 protein by 50%, with peak inclusion at 70% in embryos followed by postnatal decline. This fetal-like splicing pattern recapitulation depletes functional sodium channel proteins, providing temporal insights into the early onset of DS (Voskobiynyk et al., 2021).

Functional heterogeneity of intronic variants emerges at the molecular level: c.4853–1 G > C in intron 25 traps truncated proteins in the endoplasmic reticulum, exerting “dominant-negative effects” on membrane localization with mild functional impairment, whereas c.4853–25 T > A induces “haploinsufficiency” via pronounced decreases in current density and voltage sensitivity. This comparative analysis highlights how different positional variants within the same intron can drive phenotypic diversity through distinct mechanistic pathways, underscoring the predictive value of splicing regulation for clinical phenotypes (Hammer et al., 2022).

Molecular dissection of the splicing regulatory network expands pathogenic understanding: rare variants in intron 20 such as chr2:166864064G > A disrupt binding sites for splicing factors like SRSF1, triggering poison exon 20 N inclusion and nonsense-mediated decay of full-length mRNA. Identified in five DS and GEFS+ cases, this mechanism not only validates non-coding variants as disruptors of core RNA processing machinery but also highlights splicing regulatory elements as potential therapeutic targets for *SCN1A*-related epilepsies (Carvill et al., 2018).

Transcriptional dysregulation challenges the classical “haploinsufficiency” paradigm: homozygosity for *rs7587026* increases hippocampal *SCN1A* expression by 50%, accompanied by reduced hippocampal volume and a 6-fold increase in spontaneous seizures in zebrafish. These findings establish a dual “transcriptional activation-neuronal hyperexcitability” mechanism, demonstrating that *SCN1A*-related disorders arise from both insufficient and excessive transcriptional dosage, which disrupts neuronal electrophysiological homeostasis. This bidirectional regulation theory provides a novel framework for interpreting the continuum of phenotypes from loss-of-function to gain-of-function (Silvennoinen et al., 2022).

**Figure 1:**
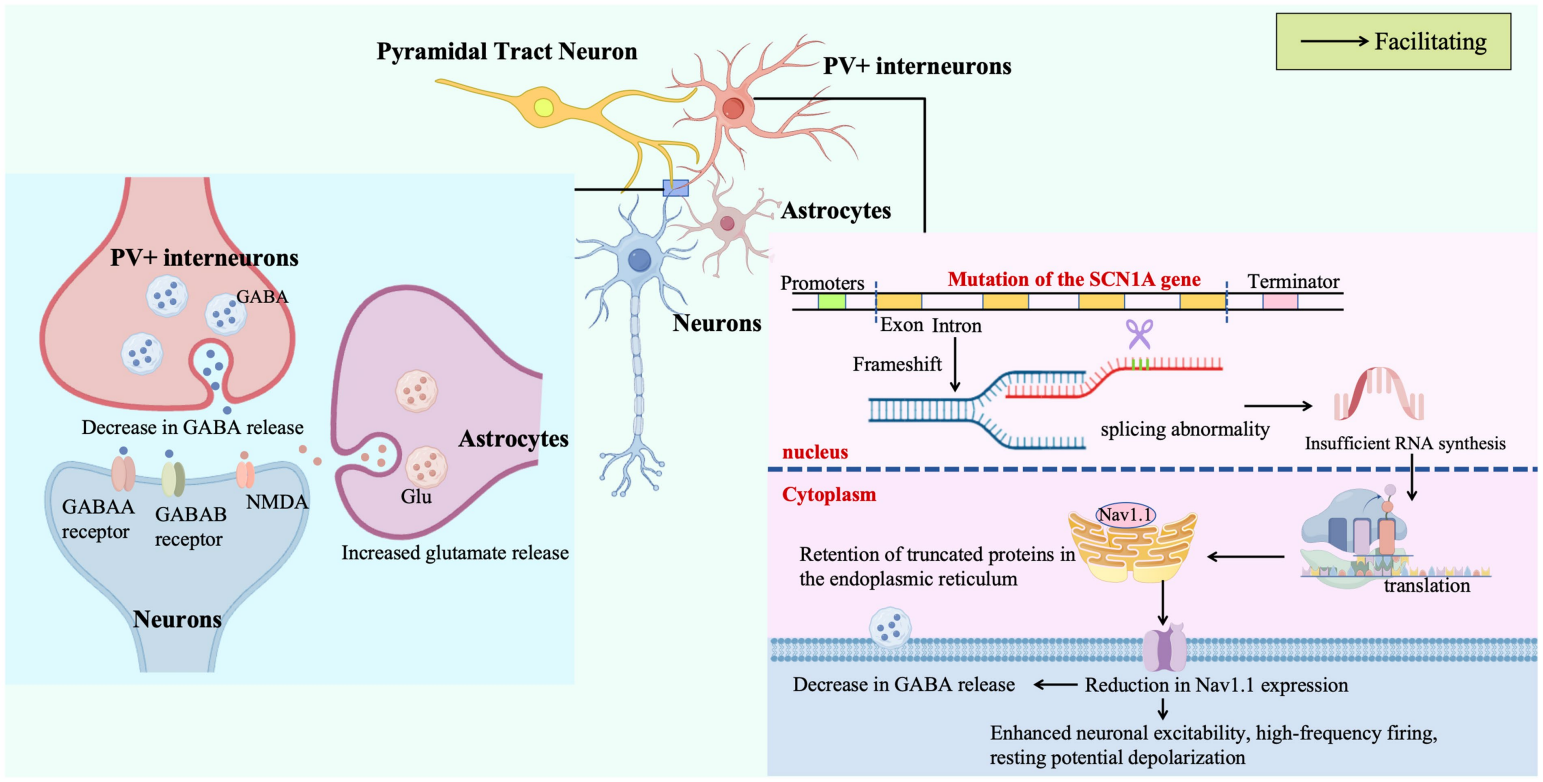
New insights into the mechanism of DS.

Non-coding variants in *SCN1A* drive phenotypic diversity via position-specific splicing anomalies (e.g., exon skipping, intron retention) and bidirectional transcriptional dysregulation (deficiency/excess), with splicing factor disruption and transcription-excitability mechanisms underpinning phenotype prediction (Figure 1). Current research lacks validation in human primary cells and clarity on transcription-splicing interactions and genome-wide non-coding variant landscapes. Future studies may use multi-omics and humanized models to dissect cell-specific effects, targeting RNA regulatory elements for therapeutic development to bridge mechanisms and clinical applications.

### 2.2 Ion Channel dysfunction and neuronal excitability dysregulation: pathogenic cascades from molecular gating to network homeostasis

Aberrant gating kinetics of Nav1.1 channels, driven by *SCN1A* missense mutations, represent a core molecular mechanism underlying inhibitory neuron dysfunction. In a patient with severe encephalopathy carrying biallelic variants p. A1685S and mosaic p. T782I, the T782I mutation induces a prominent persistent sodium current, accounting for 7.1% of peak current (*p* < 0.0003), enhancing neuronal excitability and triggering high-frequency firing independently of protein expression levels. This provides human evidence for a gain-of-function mechanism mediated by non-inactivating currents, challenging the conventional focus on haploinsufficiency (Gorman et al., 2021). Complementary findings in *SCN1A* A1783V mice show that a + 10 mV right shift in activation voltage and accelerated slow inactivation—despite preserved peak current—reduce interneuron firing frequency by 30% and increase rheobase, confirming that gating dysfunction alone can drive pathological phenotypes (Layer et al., 2021).

At the subcellular level, Nav1.1’s localization is critical for its function. Nav1.1 is primarily located in the axon initial segment of parvalbumin-positive (PV+) interneurons. It plays a critical role in the spike output of PV interneurons, and dysfunction of the PV inhibitory circuit leads to epileptic seizures in knock-in mice (Ogiwara et al., 2007). Nav1.1 channels are predominantly expressed in GABAergic interneurons (e.g., parvalbumin-positive PV, somatostatin-positive SST, and vasoactive intestinal peptide-positive VIP interneurons). Loss of Nav1.1 function reduces the intrinsic excitability of these inhibitory neurons, disrupting the brain’s excitation-inhibition balance and triggering epilepsy. In DS mouse models, augmenting Nav1.1 expression via antisense oligonucleotides or using the TrkB receptor partial agonist LM22A-4 rescues synaptic inhibition and reduces seizures (Debanne et al., 2024).

At the network level, haploinsufficiency of Nav1.1 in PV + interneurons increases the threshold for action potential generation, impairs train firing, and reduces postsynaptic potential amplification, thereby increasing susceptibility to thermally-induced seizures. Additionally, Nav1.1 deficiency in PV + interneurons specifically causes autistic-like behaviors without hyperactivity. Co-deletion of Nav1.1 in PV + and somatostatin-expressing interneurons synergistically prolongs seizure duration and impairs long-term spatial memory. This “interneuron hyperactivation-homeostatic plasticity imbalance” mechanism underscores how subtle gating changes can propagate to circuit-level dysfunction, explaining the severity of early-onset epilepsies (Rubinstein et al., 2015).

Structural and regulatory insights deepen our understanding of these mechanisms: cryo-EM structures identify mutation hotspots at the Nav1.1 voltage-sensor–pore domain interface, including T217, A223, and I227, as well as the selectivity filter sites such as E954 and D1727, where mutations disrupt electromechanical coupling or ion selectivity. Comparative analysis with Nav1.5 highlights 70 conserved mutational loci, such as T1658R in VSD-IV that shifts inactivation curves leftward, linking epilepsy and cardiac channelopathies through shared functional domains (Pan et al., 2021). Posttranslational regulation via *NEDD8* conjugation adds another layer: by protecting Nav1.1 from proteasomal degradation at K1936 in inhibitory interneurons, neddylation maintains channel stability; conditional NEDD8 deficiency reduces Nav1.1 levels by 40%, phenocopying haploinsufficiency and identifying this modification as a critical functional buffer (Cannon, 2021).

At the circuit level, Nav1.1 haploinsufficiency induces cell-type-specific excitability changes in the thalamus: excitability is impaired in glutamatergic neurons of the reticular and ventral posterolateral nuclei but enhanced in the ventral posteromedial nucleus (Studtmann et al., 2022). Mechanistically, reduced Nav1.1 expression decreases sodium current density and action potential firing frequency in GABAergic interneurons, thereby reducing GABA release and weakening inhibitory synaptic transmission, which is accompanied by increased frequency and faster kinetics of GABAergic input to ventral posterolateral neurons (Mantegazza and Broccoli, 2019). These regional imbalances in excitatory-inhibitory homeostasis reveal how ion channel defects reshape firing patterns across synapses, providing a circuit-based explanation for the characteristic rhythmic abnormalities in DS.

Missense mutations in *SCN1A* drive pathogenic cascades via Nav1.1 gating abnormalities (e.g., enhanced persistent sodium current), directly increasing excitability or indirectly disrupting network homeostasis through inhibitory interneuron/thalamic excitation-inhibition imbalances. Structural biology-defined mutational hotspots (voltage sensor-pore interfaces) and conserved cardiac sodium channel loci, alongside NEDD8-mediated channel stability regulation, explain phenotypic diversity (Figure 1). Current gaps include neuron subtype-specific mechanisms and modification-variant interactions; future studies should integrate single-cell electrophysiology with spatial transcriptomics to dissect cell-type effects and use multi-scale modeling to clarify signal transduction from channels to networks, informing excitability-targeted epilepsy therapies.

### 2.3 Neural circuitry and inhibitory synaptic dysfunction: from microcircuits to Brainwide excitation-inhibition imbalance in DS

Electrophysiological deficits in inhibitory interneurons form the core cellular basis of network imbalance in DS, with pronounced subtype specificity and developmental stage dependency. In a mouse model carrying the patient-specific *SCN1A* mutation (p. H939R), PV + interneurons in the hippocampal CA1 region exhibit depolarized resting membrane potential, reduced action potential amplitude, and decreased marker expression, collaborating with enhanced pyramidal neuron excitability to drive local network hypersynchronization; kinetic delays in sodium currents of patient iPSC-derived neurons validate *SCN1A* mutation-driven disruptions to inhibitory circuit integrity (Dyment et al., 2020). Notably, *Ndnf*-positive interneurons remain resilient during the critical developmental period (P16-21), lacking Nav1.1 expression at their axon initial segments (AIS), which highlights heterogeneous vulnerability among inhibitory neuron subtypes (Voskobiynyk and Paz, 2024). In contrast, PV + fast-spiking interneurons (PVINs) show transient dysfunction at P18-21—reduced firing frequency, impaired high-frequency discharge, and compensatory axon initial segment (AIS) elongation—normalizing by P35, suggesting early-life inhibitory neuron impairment triggers childhood seizures while postnatal compensation contributes to chronicity (Favero et al., 2018).

Synaptic transmission abnormalities exacerbate excitation-inhibition (E-I) dysbalance across spatial and temporal scales. Hippocampal-specific *SCN1A* deletion reduces the frequency of spontaneous inhibitory postsynaptic currents (sIPSCs) in dentate granule cells without altering excitatory inputs, directly leading to thermosensitive seizures and spatial memory deficits, which establishes inhibitory input deficiency in hippocampal microcircuits as a neurobiological basis for DS comorbidities (Stein et al., 2019). At the synaptic terminal, reduced vesicular release probability, fewer inhibitory synapses, and impaired calcium signaling (failure of high extracellular calcium to enhance IPSC amplitude) disrupt E-I balance at the molecular level (Uchino et al., 2021). In zebrafish, Scn1Lab deficiency increases excitatory synapses (PSD-95), reduces inhibitory synapses (gephyrin), and elevates excitatory neuron apoptosis, providing evolutionary conserved evidence for synaptic homeostasis disruption driving early network hyperexcitability (Brenet et al., 2024).

Dynamic remodeling of neural circuits represents a key mechanism for seizure initiation and propagation, with distinct regional and network-level characteristics. Excessive excitatory input from the entorhinal cortex to dentate granule cells, rather than local PV + interneuron dysfunction, underlies dentate gyrus hyperexcitability in DS mice; optogenetic activation of entorhinal cortex lowers seizure thresholds, while chemogenetic inhibition of dentate PV + cells exacerbates seizures, identifying excessive excitatory drive in corticohippocampal circuits as a core pathogenic pathway (Feng and Shuman, 2022). Computational models reveal that *SCN1A* mutations reduce the critical threshold for E-I synaptic gains by decreasing inhibitory interneuron excitability or increasing firing thresholds, promoting transitions from low-amplitude asynchronous to high-amplitude γ-band synchronized activity— a hallmark of network criticality dysregulation conserved across DS models (Du et al., 2019). Thalamic circuit analysis uncovers cell-type-specific excitability changes: Nav1.1 haploinsufficiency impairs nRT/VPL glutamatergic neuron excitability but enhances VPM excitability, with accelerated GABAergic input kinetics to VPL neurons, providing circuit-level insights into characteristic thalamocortical rhythm abnormalities (Studtmann et al., 2022).

Genetic modifiers and receptor dysfunction in the GABAergic system constitute deep molecular regulatory mechanisms. The Dsm1 locus on mouse chromosome 5 identifies Gabra2 as a key modifier gene, whose expression correlates with thermosensitive seizure susceptibility and survival in DS mice; clobazam targeting Gabra2 elevates seizure thresholds dose-dependently by enhancing GABAergic signaling, linking background genetics to *SCN1A* haploinsufficiency (Hawkins et al., 2016). Dravet-associated mutations in *GABRA1, GABRB2,* and *GABRG2* converge on disrupting α1β2γ2 GABAA receptor function—GABRA1/GABRB2 impair gating kinetics, while GABRG2 disrupts receptor trafficking—thereby compromising inhibitory synaptic transmission efficiency (Hernandez et al., 2021). Temporal dynamics in hippocampal microcircuits reveal stage-dependent E-I remodeling: during the severe stage, CA1 Oriens interneurons exhibit elevated action potential thresholds and reduced synaptic firing, whereas pyramidal neurons transition from transient hyperexcitability (pre-epileptic stage) to decreased excitability, reflecting adaptive rewiring of microcircuit homeostasis (Almog et al., 2021).

Multi-circuit collaborative pathogenicity is particularly evident in limbic systems: synaptic abnormalities in the bed nucleus of the stria terminalis (BNST)—increased sEPSC amplitude, reduced sIPSC frequency, and elevated AMPA/NMDA ratios—may contribute to respiratory dysfunction and sudden death in DS; concurrent reductions in cortical inhibitory neuron calcium activity and GABA concentration (Takado et al., 2022) form trans-cerebral cascades with hippocampal and thalamic circuit abnormalities, driving a vicious cycle of network hyperexcitability. Local ablation experiments confirm that Nav1.1 dysfunction in either hippocampus or cortex is sufficient to trigger epileptic networks, underscoring the multi-regional origin of seizure propagation in DS (Jansen et al., 2020).

In DS, inhibitory synaptic dysfunction disrupts microcircuit homeostasis via electrophysiological abnormalities (depolarized resting potential, reduced action potential amplitude) in hippocampal parvalbumin-positive interneurons and deficits in inhibitory synaptic transmission, with excessive excitatory drive across brain circuits and genetic modifiers of the GABAergic system exacerbating brainwide excitation-inhibition imbalance (Figure 1). Current research lacks understanding of subtype-specific developmental heterogeneity in inhibitory neurons and interactions between synaptic remodeling and ion channel dysfunction. Future studies should use single-cell approaches to map cell-type specific pathogenic pathways and employ multi-scale modeling alongside GABAA receptor-targeted strategies to address abnormal network synchronization in epilepsy.

### 2.4 Genetic heterogeneity and multi-system interactions: decoding complex pathogenesis beyond *SCN1A* monogenic defect

The phenotypic diversity of DS arises from intricate interactions between *SCN1A* mutations, polygenic backgrounds, non-neuronal cell dysfunction, and systemic regulatory abnormalities. Exome sequencing identifies rare variants in epilepsy-associated genes (e.g., *DEPDC5*, *CHD2*) enriched in DS patients, with *SCN1A-DEPDC5* co-mutations often associated with focal cortical dysplasia. These findings suggest collaborative disruption of neurodevelopmental pathways (e.g., mTOR signaling, chromatin remodeling), where polygenic variants amplify *SCN1A*-mediated sodium channel dysfunction. The paradox of reduced intelligence-related vs. increased longevity-related polygenic risk scores further implies genomic background modulates disease trajectories through systemic metabolic or immune pathways, transcending the traditional view of DS as a pure Nav1.1 channelopathy (Martins Custodio et al., 2023).

Arachidonic acid metabolism provides a molecular bridge for polygenic synergy: *ALOXE3* promoter and missense variants disrupt TFII-I binding and catalytic activity, respectively, leading to dysregulated lipid mediator production and enhanced neuronal membrane excitability. This metabolic-electrophysiological coupling anomaly not only explains pharmacoresistance in subset patients but also identifies ALOXE3 as a genetic modifier of *SCN1A* function, potentially through membrane lipid homeostasis regulation (Gao et al., 2021). Convergent dysfunction of GABAA receptor subunits (*GABRA1/2/3*) forms a pathogenic hub: GABRA1/GABRB2 variants impair channel gating, while GABRG2 disrupts receptor trafficking, collectively reducing inhibitory synaptic currents. This cross-subunit vulnerability validates the hypothesis of “inhibitory system fragility” in multi-genic backgrounds, highlighting α1β2γ2 receptors as druggable nodes for combinatorial deficits (Hernandez et al., 2021).

Epigenetic network dysregulation reveals upstream effects of *SCN1A* mutations: patient-derived GABAergic neurons exhibit hyperactivated chromatin remodeling genes (e.g., histone methyltransferases) and cell cycle regulators (*FOXM1, E2F1*), leading to dysregulated neurodevelopmental gene expression and oxidative stress hypersensitivity. This epigenetic reprogramming not only impairs inhibitory neuron differentiation but also propagates transgenerational gene expression abnormalities, providing an epigenetic framework for DS neurodevelopmental delays (Schuster et al., 2019).

Astrocytic calcium signaling dysfunction marks a paradigm shift toward neuroglial interactions: *Scn1a+/−* mice show increased frequency (but unchanged amplitude) of astrocytic calcium oscillations and enhanced ATP (adenosine triphosphate)-evoked calcium influx, indicating glial cells modulate neuronal excitability via abnormal release of gliotransmitters (e.g., glutamate, ATP). This “astrocyte-neuron” signaling loop creates a positive feedback mechanism—neuronal excitotoxicity exacerbates glial calcium dysregulation, which in turn amplifies network hypersynchronization through gap junctions—challenging the neuron-centric pathogenesis model (Uchino et al., 2023).

Astrocytic calcium signaling dysfunction marks a paradigm shift toward neuroglial interactions: *Scn1a+/−* mice show increased frequency (but unchanged amplitude) of astrocytic calcium oscillations and enhanced ATP-evoked calcium influx, indicating glial cells modulate neuronal excitability via abnormal release of gliotransmitters (e.g., glutamate, ATP). Concomitantly, this calcium dysregulation disrupts the astrocyte-neuron lactate shuttle (ANLS), a metabolic axis where astrocytic glycolysis generates lactate that is shuttled to neurons via monocarboxylate transporters (MCT1/MCT4 in astrocytes; MCT2 in neurons) to sustain oxidative phosphorylation (Uchino et al., 2023; Bonvento and Bolaños, 2021). Impaired ANLS in *SCN1A*-deficient models reduces neuronal lactate uptake, forcing reliance on inefficient oxidative metabolism and exacerbating energy deficits (Yang et al., 2024). This “astrocyte-neuron” signaling loop creates a dual-pathway positive feedback mechanism—neuronal excitotoxicity exacerbates glial calcium dysregulation, which in turn amplifies network hypersynchronization through gap junctions, while concurrent ANLS disruption compromises neuronal bioenergetics. Specifically, astrocytic calcium oscillations normally promote glycolytic flux and lactate release (Bonvento and Bolaños, 2021), but in *Scn1a+/−* cells, dysregulated calcium signaling impairs lactate production and shuttling, leading to reduced neuronal ATP availability and compromised regulation of voltage-gated ion channels (e.g., *CACNA1A, KCNJ2*; Yang et al., 2024). This metabolic-ionic coupling defect challenges the neuron-centric pathogenesis model, as glial metabolic dysfunction directly propagates excitability abnormalities via both neurotransmitter-mediated signaling and energy substrate deprivation.

Collapse of energy metabolism-ion channel coupling represents a core downstream effect of *SCN1A* deficiency: transcriptomic analysis reveals downregulation of glycolytic enzymes (HK2, PFKL) and transcriptional reprogramming of calcium channels (CACNA1A, KCNJ2) in *SCN1A-KO* cells, forcing neurons to rely on inefficient oxidative phosphorylation. Hippocampal metabolomics corroborates this with reduced TCA cycle intermediates and glutamate/GABA imbalance, providing mechanistic rationale for ketogenic diet efficacy via ketone-mediated energy rescue and neurotransmitter modulation (Shi et al., 2019; Miljanovic et al., 2021).

Metabolomic profiling has further identified alterations in specific lipid mediators, such as monoacylglycerols, during seizure states in DS models, suggesting potential roles in epileptogenesis beyond classical energy metabolism (Bahceci et al., 2023).

Neurovascular-immune interactions uncover novel disease progression pathways: BBB leakage (ZO-1 reduction, IgG extravasation) activates innate immune responses in the brain, with serum-derived inflammatory factors—rather than immune cell infiltration—exacerbating neuronal apoptosis via the VHL/HIF-1α/p21 axis. *SCN1A* deficiency suppresses VHL expression, inducing HIF-1α accumulation and p21-mediated cell cycle arrest, a paradoxical mechanism where overactivated neuroprotective pathways lead to hippocampal neuron loss (Alonso et al., 2025; Kong et al., 2024).

Serotonin signaling rewiring offers precision therapy opportunities: zebrafish studies show 5-HT₂B receptor activation enhances GLT-1-mediated glutamate clearance by regulating astrocytic transporter trafficking, linking *SCN1A*-induced glutamatergic dysregulation (GLT-1 downregulation) to serotonin system modulation. This mechanistic insight justifies clinical trials of serotonin-targeting drugs (e.g., fenfluramine), proposing multi-target interventions on neurotransmitter transporters/receptors as a strategy to overcome limitations of sole sodium channel targeting (Griffin et al., 2019; Hameed et al., 2023).

Phenotypic diversity in DS arises from interactions between *SCN1A* mutations, polygenic backgrounds (e.g., DEPDC5, ALOXE3), epigenetic dysregulation (activated chromatin remodeling genes), and neuroglial/metabolic system perturbations: polygenic variants disrupt mTOR signaling, GABA receptor function, and arachidonic acid metabolism, while astrocytic calcium dysregulation amplifies network excitability via gliotransmitter signaling. Energy metabolism-ion channel collapse and neurovascular-immune interactions further exacerbate pathology at metabolic and microenvironmental levels. Current research lacks understanding of molecular hubs mediating multi-system crosstalk (e.g., *ALOXE3*-driven metabolic-electrophysiological coupling) and cell-type specific defects (e.g., astrocyte-neuron synergy) (Figure 1). Future studies should use single-cell multi-omics to identify key interaction nodes and integrate multi-modal data to construct multi-system pathogenic networks, informing precision therapies targeting polygenic pathways and neuroglial interactions.

Missense mutations in *SCN1A* disrupt pre-mRNA splicing, leading to inadequate mRNA synthesis and accumulation of truncated, misfolded proteins within the endoplasmic reticulum. This impairs the trafficking and plasma membrane expression of Nav1.1 sodium channels in PV + inhibitory interneurons, critical regulators of neuronal excitability. The resulting Nav1.1 deficiency in PV + cells drives electrophysiological dysfunction, including heightened excitability, high-frequency firing, and resting membrane potential depolarization, which collectively reduce the release of the inhibitory neurotransmitter γ-aminobutyric acid (GABA). Concurrently, astrocytes exhibit aberrant release of adenosine triphosphate (ATP) and the excitatory neurotransmitter glutamate, perpetuating a pathological cycle of sustained neuronal hyperexcitability and synchronous discharges that manifest as drug-resistant seizures and other clinical hallmarks of DS.

## 3 Diagnosis: a comprehensive framework from clinical phenotype analysis to molecular precision testing

### 3.1 Key indicators for clinical phenotypic characteristics and early identification

DS, a voltage-gated sodium channelopathy caused by *SCN1A* dysfunction, requires dynamic integration of early epileptic phenotypes and neurodevelopmental trajectories for clinical diagnosis. Classic cases present with febrile seizure susceptibility before 6 months of age, characterized by fever-induced generalized tonic–clonic seizures (FS+), with 83% developing afebrile seizures such as focal-to-generalized seizures and progressive motor delay GMFM-88 score decline during follow-up (Li et al., 2021; Fan et al., 2023). The International League Against Epilepsy (ILAE 2023) criteria emphasize constructing a multidimensional diagnostic matrix using seizure-evoked potential (SEP) monitoring for cortical hyperexcitability onset age <12 months in 91% seizure type evolution 67% hemiclonic seizures and non-epileptic symptoms such as language stagnation after 18 months (Solaz et al., 2024; Nordli 3rd et al., 2024).

A specific early marker is rhythmic eyelid stereotypies 3–5 closures/s in 20% of children at 12–24 months, mediated by brainstem reticular formation dysregulation. Video-EEG shows no epileptiform discharges and these behaviors are transiently suppressible by tactile stimulation distinguishing DS from myoclonic epilepsies like Lennox–Gastaut syndrome (Sala-Coromina et al., 2021). Standardized parental observation using the ISISS seizure record increases fever-seizure association recognition by 40% enabling timely initiation of sodium channel modulators such as stiripentol within the first year of illness (Soto Jansson et al., 2024).

While the current multidimensional diagnostic framework based on *SCN1A* dysfunction has notably improved the early identification of DS, its sensitivity in atypical phenotypic cases still requires validation through expanded prospective cohort studies. Integrating single-cell sequencing to dissect the developmental trajectories of sodium channelopathy-related neural circuits in the future may facilitate a paradigm shift from symptom-driven diagnosis to mechanism-oriented intervention.

### 3.2 Innovations in molecular diagnosis for precision application

*SCN1A* genetic testing follows a three-tiered pathway—mutation spectrum analysis functional prediction inheritance pattern validation—to establish core biological evidence. Targeted sequencing covering 28 exons and ±50 bp splice sites average depth 1,000 × identifies pathogenic variants with hotspots like c.3772C > T p. Arg1258Trp accounting for 34% and *de novo* mutations comprising 65.75% (Giorgi et al., 2024; Mahdieh et al., 2018). For variants of uncertain significance (VUS) a Bayesian network integrating 13 in silico tools MutationTaster 2.0 training set n = 5,238 CADD ≥20 for deleterious variants reclassifies 56.5% of VUS: 17.8% as pathogenic 38.7% as likely pathogenic revising recurrence risks from 5 to 25% in mosaic mutation families (Gonsales et al., 2019; Xu et al., 2015).

A multicenter validation cohort n = 1,018 demonstrates that a genetic risk score model Score = 0.6 × pathogenicity score+0.4 × 1/onset age in months achieves an AUC of 0.89 95%CI: 0.86–0.92 reducing misdiagnosis rates by 11% compared to single markers such as onset age AUC = 0.74 for differentiating DS from GEFS+ (Brunklaus et al., 2022). Technological advancements include portable nanopore sequencing (MinION) which achieves 98.7% concordance for missense mutations such as p. L1630P in <4 h at $291 per sample though sensitivity for INDELs is 72% necessitating MLPA for copy number variant validation (Ngo et al., 2021).

While the current three-tiered *SCN1A* molecular diagnosis system enhances variant interpretation accuracy through Bayesian networks and genetic risk scoring, its missed detection rate in rare splice-site variants and mosaic mutations still requires calibration with multicenter data. Integrating spatial transcriptomics to characterize tissue-specific expression effects of mutation sites in the future may advance molecular diagnosis toward a precision medicine model enabling dynamic risk prediction and treatment response forecasting.

### 3.3 Integrated diagnostic value of multimodal auxiliary examinations

Neuroimaging and electrophysiology provide pathophysiological corroboration for molecular diagnosis. 3 T MRI volumetry reveals hippocampal volume reduction in 18.6% of patients standardized mean difference −1.23 ± 0.37 *p* < 0.01 correlated with seizure frequency r = 0.42 *p* = 0.003 and WPPSI language quotient r = −0.35 *p* = 0.011 implicating hippocampal dysfunction in cognitive impairment (Ventura et al., 2024). Electrophysiological phenomics uses 92 microelectrode array-derived features *θ*/*δ* power ratio spike conduction velocity etc. to train a Gaussian Naïve Bayes classifier training n = 156 validation n = 72 with 80.77% accuracy in predicting *SCN1A* mutation classes—loss-of-function mutations show significantly reduced γ-band power density 30–100 Hz *p* < 0.001 establishing an “electrophysiological fingerprint-genotype” mapping (Kress et al., 2022).

Future diagnostic advancements may leverage non-invasive imaging modalities beyond conventional MRI, such as optical coherence tomography (OCT), which holds promise for capturing glial dynamics in the retina as potential biomarkers reflecting central nervous system pathology (Salazar et al., 2023).

Following ACMG/AMP 2015 guidelines a multimodal evidence framework inheritance pattern functional assays population frequency gnomAD allele frequency <0.001 increases VUS interpretability from 43.5 to 82.3% providing robust support for prenatal diagnosis such as chorionic villus sampling (Gonsales et al., 2019).

Multimodal auxiliary examinations have established a genotype–phenotype correlation framework through neuroimaging quantification and electrophysiological phenomics, yet their diagnostic utility in early subclinical stages is limited by the nonspecificity of hippocampal structural changes and developmental stage-dependent variability of electrophysiological features. Future integration of cross-omics data to develop dynamic assessment models may drive auxiliary examinations from diagnostic evidence integration to functional transformation for therapeutic target discovery.

### 3.4 Lifecycle diagnostic optimization and complex case management

Diagnosis of adult and atypical DS relies on genetic resolution of phenotypic variability. Retrospective analysis identifies *SCN1A* pathogenic variants in 0.74% of adult refractory epileptics median age 44.5 years presenting with drug-resistant tonic–clonic seizures 89% and progressive pyramidal signs 60% hyperreflexia without childhood febrile seizure clusters (Silvennoinen et al., 2022). Electrophysiological studies show voltage-dependent inactivation curves shifted rightward V1/2 = −22.3 ± 1.8 mV vs. − 26.7 ± 2.1 mV in controls explaining a 37% seizure frequency increase with sodium channel blockers such as carbamazepine due to enhanced membrane excitability (Feng et al., 2024).

A dynamic genetic scoring system incorporating mutation pathogenicity seizure control time baseline language quotient from a 10-year cohort shows that scores >110 predict a 5-year seizure-free rate of 12% versus 38% in low-score groups *p* = 0.008. Integrated into clinical decision support systems (CDSS) this model generates real-time diagnostic confidence reports by fusing genetic results such as *SCN1A* functional classification EEG spectral data θ-band abnormalities MRI hippocampal volume—recommending stiripentol-cannabidiol for children confidence ≥90% and screening for *SCN2A/GABRG2* co-mutations in adults confidence <70% thus enabling lifespan diagnostic management (Feng et al., 2024).

The lifecycle diagnostic framework for adult and atypical DS has achieved cross-age management breakthroughs through dynamic genetic scoring and clinical decision systems, yet its diagnostic efficacy in complex phenotypic variants remains constrained by age-specific biases in genotype–phenotype mapping. Future integration of longitudinal multi-omics tracking and artificial intelligence algorithm optimization may drive diagnostic models from stage-specific risk assessment to full-course precision intervention strategy generation.

## 4 Traditional therapies: current standards and unmet needs

The management of DS is inherently challenging as conventional antiepileptic drugs (AEDs) primarily aim to reduce seizure frequency rather than achieve elimination, a limitation rooted in the high prevalence of pharmacoresistance driven by *SCN1A*-mediated network hyperexcitability (de Lange et al., 2018; Wallace et al., 2016). Clinical guidelines advocate a stratified treatment approach (Table 1; Strzelczyk, 2022).

**Table 1:** Overview of main traditional therapeutic approaches for DS.

Category	Mechanism of action	Representative agent/intervention	Key characteristics/notes
1st Line	Broad-spectrum voltage-gated sodium channel modulator	Valproic Acid (VPA) (de Lange et al., 2018; Strzelczyk, 2022; Wallace et al., 2016)	Broad-spectrum antiseizure activity; used for early-onset seizures; note hepatotoxicity and teratogenicity risks
	Potentiates GABAA receptor-mediated inhibition	Clobazam (CLB) (Strzelczyk, 2022; Wallace et al., 2016)	Benzodiazepine; often used with VPA; note respiratory depression risk at high doses or with opioids
2nd Line	Cytochrome P450 enzyme inhibitor (primarily increases CLB)	Stiripentol (STP) (Strzelczyk, 2022; Zulfiqar Ali et al., 2020)	Requires combination with CLB; significantly increases CLB plasma concentration (2–3 fold); note sedation risk and potential hyperammonemia (especially with VPA).
	Targets endocannabinoid system (e.g., CB1 receptor mod.)	Cannabidiol (CBD) (Strzelczyk, 2022; Devinsky et al., 2019; Devinsky et al., 2017; McNamara et al., 2020)	FDA-approved for DS; note risk of hepatic transaminase elevation (esp. with VPA) and thrombocytopenia risk with VPA co-administration
	Modulates 5-HT2B receptor-mediated inhibitory neural tone	Fenfluramine (FFA) (Strzelczyk, 2022; Schoonjans, 2021; Schoonjans, 2019)	Significantly reduces convulsive seizure frequency; serotonergic activity; historical use in obesity (high doses linked to VHD, not reported at low doses in epilepsy); may exacerbate VPA-induced thrombocytopenia
Adjunctive	Blocks Na+/Ca2 + VG channels, enhances GABA, inhibits AMPA rec.	Topiramate (TPM) (Strzelczyk, 2022; Brandt et al., 2015)	Multiple MOAs; limited efficacy; note cognitive adverse effect risk (esp. in patients with ID)
	Enhances GABAergic inhibition (possibly via Cl-homeostasis mod.)	Bromide (Strzelczyk, 2022)	Historical agent; limited use, limited efficacy
Contraind	Enhances voltage-gated Na + channel inactivation	Carbamazepine, Oxcarbazepine, etc. (de Lange et al., 2018; Strzelczyk, 2022; Wallace et al., 2016)	Contraindicated: Significantly increases risk of status epilepticus & seizure frequency by
Non-Pharm	Modulates brain energy metabolism (ketones: antiseizure)	Ketogenic Diet (Strzelczyk, 2022)	High-fat, low-carb diet; effective in some patients, requires strict selection and management
	Stimulates vagus nerve to inhibit cortical hyperexcitab.	Vagus Nerve Stimulation (VNS) (Strzelczyk, 2022)	Implanted device; effective as adjunctive therapy in some cases
Category	Mechanism of Action	Representative Agent/Intervention	Key Characteristics/Notes

Table focuses on pharmacological mechanisms and key characteristics. Detailed toxicities and interactions are in Table 2.

First-line therapy: Valproic acid (VPA) and clobazam (CLB) serve as cornerstones, exerting broad-spectrum sodium channel modulation and potentiating GABAergic inhibition to control early-onset seizures, respectively (de Lange et al., 2018; Strzelczyk, 2022; Wallace et al., 2016).

Second-line therapy: For patients with inadequate response to first-line agents, options include stiripentol (STP, enhancing CLB efficacy), cannabidiol (CBD, targeting endocannabinoid signaling), and fenfluramine (FFA, regulating 5-HT2B receptor-mediated inhibitory tone; Strzelczyk, 2022; Devinsky et al., 2019; Ng et al., 2011; Schoonjans, 2019; Table 2).

**Table 2:** Major challenges and limitations of traditional pharmacotherapy in DS.

Challenge Type	Specific Problem & Risk	Primary Agents/Combinations	Key Reason/Consequence/Note
Drug Toxicity	Severe hepatotoxicity risk	VPA (Strzelczyk, 2022; Wallace et al., 2016)	High risk in young children
	Increased teratogenic risk (neural tube defects)	VPA (Bromfield et al., 2008)	Odds Ratio (OR) = 2.3; extreme caution in women of childbearing potential
	Respiratory depression risk	CLB (Strzelczyk, 2022)	Associated with high doses or opioid co-administration (risk 3–5%)
	Hepatic transaminase elevation	CBD (esp.with VPA) (Devinsky et al., 2019)	~17.2% incidence in VPA co-administered patients
	Thrombocytopenia risk	VPA + CBD combination (McNamara et al., 2020)	~1/3 of co-administered patients affected (risk significantly higher than monotherapy)
	Hyperammonemia risk	STP(esp. with VPA) (Zulfiqar Ali et al., 2020)	High incidence (77%) in adult DS patients
Drug Interactions	Pharmacokinetic interactions increase adverse event risk	STP + CLB (Strzelczyk, 2022)	STP inhibits CYP enzymes, leading to substantially increased CLB concentrations (2–3 fold), which increases sedation risk.
		CBD + CLB (Strzelczyk, 2022)	Bidirectional interaction increases concentrations of 7-OH-CBD and norclobazam, increasing somnolence risk.
	Shared metabolism pathways exacerbating specific toxicity	FFA may exacerbate VPA thrombocytopenia (Schoonjans, 2021)	Pharmacokinetic interaction via shared CYP450 metabolism (case report support)
Core Gap	Lack of agents directly targeting *SCN1A*/Nav1.1 dysfunction	All current therapies (de Lange et al., 2018; Wallace et al., 2016; Schoonjans, 2019)	Fundamental limitation: Failure to restore GABAergic interneuron function impaired by Nav1.1 deficits
	Low sustained seizure freedom rat	de Lange et al. (2018)	60–70% patients experience febrile/myoclonic seizures beyond early childhood

Adjunctive/Alternative therapies: Adjunctive agents like topiramate (TPM) or bromide offer limited benefit, while drugs such as carbamazepine are contraindicated due to seizure exacerbation risk (de Lange et al., 2018; Strzelczyk, 2022; Brandt et al., 2015; Wallace et al., 2016). Non-pharmacological interventions like the ketogenic diet and vagus nerve stimulation provide supplementary relief in selected cases (Strzelczyk, 2022).

This structured approach, however, is undermined by three interrelated challenges:

1. Mechanism-specific drug toxicities limiting use:

VPA: Risk of severe hepatotoxicity in young children (Strzelczyk, 2022; Wallace et al., 2016); Significantly elevated teratogenic risk (neural tube defects, OR = 2.3) in women of childbearing potential (Bromfield et al., 2008).

CLB: Increased risk of respiratory depression (3–5%) with high doses or opioid co-administration (Strzelczyk, 2022).

CBD (esp. with VPA): Risk of hepatic transaminase elevation (approx. 17.2% in VPA co-administered patients; Devinsky et al., 2019); VPA + CBD combination significantly increases thrombocytopenia risk (approx. 1/3 of patients; McNamara et al., 2020).

STP (esp. with VPA): High risk of hyperammonemia in adult patients (77%; Zulfiqar Ali et al., 2020).

2. Pharmacokinetic interactions induced by polytherapy:

STP significantly increases CLB concentrations (2–3 fold): Increases risk of sedation and somnolence (Strzelczyk, 2022).

CBD-CLB interaction: Bidirectional; increases concentrations of 7-OH-CBD and norclobazam, increasing somnolence risk (Strzelczyk, 2022).

FFA may exacerbate VPA-induced thrombocytopenia: Shared cytochrome P450 metabolism (Schoonjans, 2021).

3. Persistent critical therapeutic gap:

Lack of agents directly targeting *SCN1A* dysfunction/Nav1.1 channelopathy: No current drug corrects the impaired GABAergic interneuron function caused by NaV1.1 channel deficits (de Lange et al., 2018; Wallace et al., 2016; Schoonjans, 2019).

Consequence: 60–70% of patients continue to experience febrile and myoclonic seizures beyond early childhood (de Lange et al., 2018).

These limitations highlight the pressing need to shift from empiric, symptom-based strategies to mechanistic interventions. While existing therapies offer modest seizure control (de Lange et al., 2018; Strzelczyk, 2022; Devinsky et al., 2019; Wallace et al., 2016; Devinsky et al., 2017; Schoonjans, 2019), their off-target effects (de Lange et al., 2018; Strzelczyk, 2022; Brandt et al., 2015; Wallace et al., 2016) and inability to correct *SCN1A*-mediated channel dysfunction (de Lange et al., 2018; Wallace et al., 2016; Schoonjans, 2019) underscore the urgency for precision approaches such as gene therapy, RNA splicing correction, or selective Nav1.1 activators. Such innovations hold promise to redefine treatment by directly addressing the molecular pathogenesis of DS (Schoonjans, 2019), potentially improving long-term outcomes while minimizing the systemic toxicities inherent in current polypharmacological regimens.

These limitations highlight the pressing need to shift from empiric, symptom-based strategies to mechanistic interventions. While existing therapies offer modest seizure control, their off-target effects and inability to correct *SCN1A*-mediated channel dysfunction underscore the urgency for precision approaches such as gene therapy, RNA splicing correction, or selective Nav1.1 activators. Such innovations hold promise to redefine treatment by directly addressing the molecular pathogenesis of DS, potentially improving long-term outcomes while minimizing the systemic toxicities inherent in current polypharmacological regimens.

## 5 Precision treatment framework: from molecular targeting to neural circuit remodeling via multidimensional interventions

### 5.1 Ion channel-targeted therapy: from single-channel modulation to network homeostasis restoration

**Figure 2:**
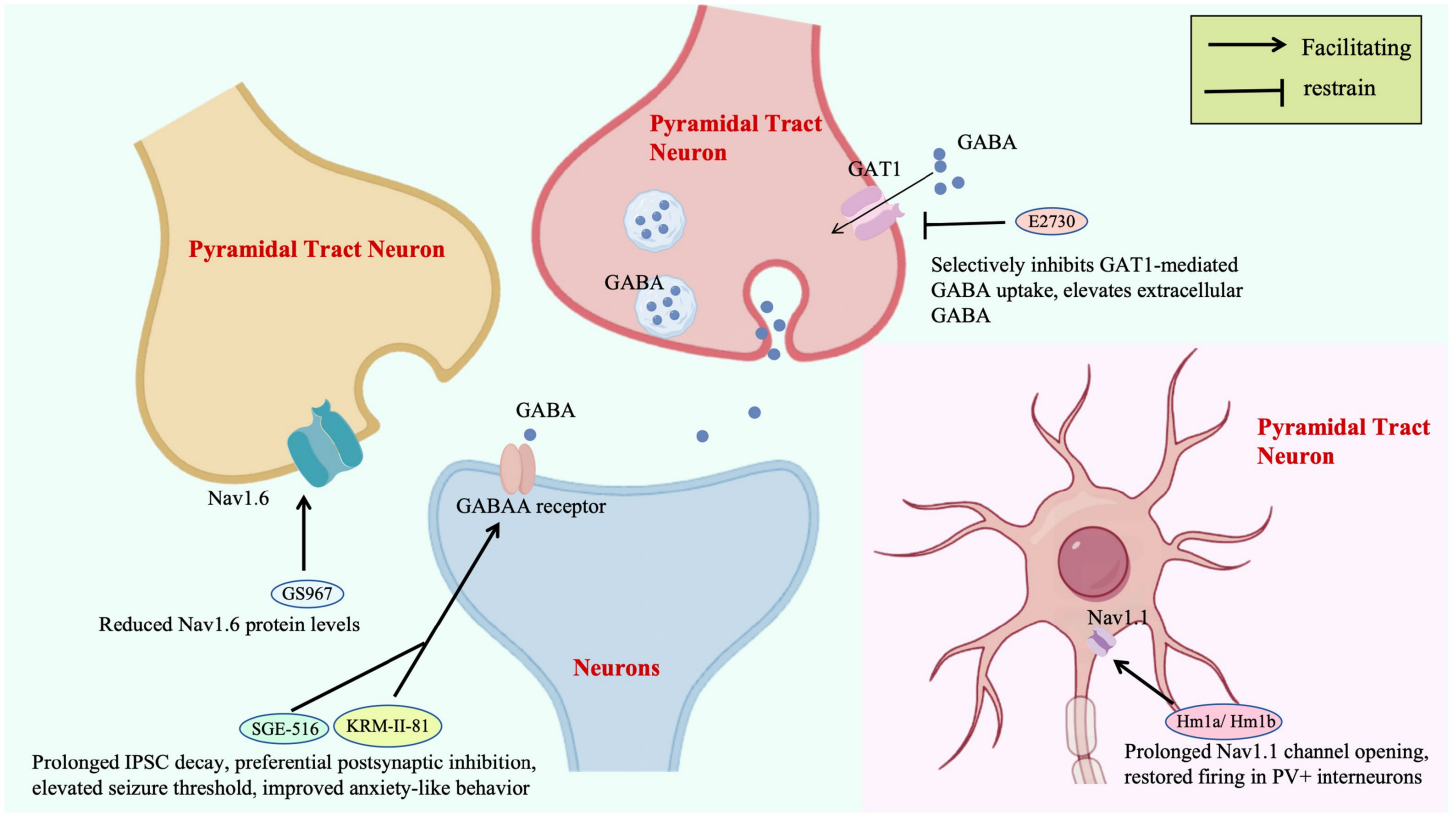
Therapeutic targets of ion channels.

Ion channel-targeted therapies for DS are evolving from single-molecule intervention to neural circuit remodeling. The spider venom peptide Hm1a selectively activates Nav1.1, restoring action potential firing in inhibitory interneurons of *Scn1a+/−* mice without affecting excitatory neurons, improving 3-day survival from 0 to 90%. This precision strategy hinges on stabilizing voltage sensor domains, providing a mechanistic template for compensating Nav1.1 dysfunction (Richards et al., 2018). Notably, Nav1.1 is highly expressed in the AIS of GABAergic interneurons, a critical site for action potential initiation (Claes et al., 2001). Hm1a binds to the voltage sensor domain of Nav1.1, stabilizes the outward movement of the S4 segment, and restores sodium current density at the AIS, thereby correcting the firing abnormalities of interneurons (Catterall, 2010). The highly selective derivative rHm1b (EC_50_ = 12 nM), by inhibiting fast inactivation and achieving a CSF half-life >70 h, surpasses its predecessor in stability and sustained efficacy, emerging as a robust therapeutic candidate (Chow et al., 2020; Figure 2).

Subtype-specific sodium channel modulators address limitations of traditional drugs: GS967 selectively reduces Nav1.6 protein levels in excitatory pyramidal neurons, suppressing hippocampal aberrant firing without affecting inhibitory interneurons. This contrasts with lamotrigine-induced seizure exacerbation, validating the safety advantage of “excitatory neuron-selective regulation” (Anderson et al., 2017; Figure 2). In zebrafish models, the combination of Nav1.1 activator AA43279 and Nav1.6 inhibitor MV1312 restores excitatory-inhibitory balance across species, establishing a cross-species paradigm for subtype-specific drug development (Weuring et al., 2020).

GABA-based therapy for epilepsy aims to enhance GABAergic neurotransmission, restoring the excitatory-inhibitory balance in neuronal networks to control seizures. As the primary inhibitory neurotransmitter, GABA binds to postsynaptic *GABAA* receptors, ligand-gated ion channels that mediate Cl^−^ influx, leading to neuronal hyperpolarization and reduced excitability, thus suppressing abnormal discharges and blocking seizure propagation in the mature brain (Perucca et al., 2023; Bryson et al., 2023). It also modulates the permeability of K^+^ and HCO_3_^−^, further influencing membrane potential and excitability to stabilize neuronal activity (Bryson et al., 2023). At presynaptic sites, GABA binds to autoreceptors to inhibit its own excessive release, a negative feedback mechanism that maintains extracellular GABA homeostasis, prevents over-inhibition, and ensures precise neurotransmission (Bryson et al., 2023). Additionally, GABA indirectly regulates neuronal excitability by affecting the release of neurotransmitters like glutamate, maintaining the balance of the brain’s neurotransmitter system (Treiman, 2001). During brain development, GABA influences neuronal migration, differentiation, and synaptogenesis; modulating GABAergic transmission may correct neurodevelopmental abnormalities in epilepsy, improving network function and enhancing seizure resistance (Bryson et al., 2023). Moreover, GABA participates in neural plasticity, facilitating post-seizure self-repair and reducing long-term functional impacts on the brain.

GABAA receptor targeting enhances inhibitory synaptic transmission through multi-dimensional mechanisms: KRM-II-81, a positive allosteric modulator of α2/3 receptors, prolongs IPSC decay by 40% to preferentially strengthen postsynaptic inhibition, elevating seizure thresholds and improving anxiety-like behavior. Its efficacy directly correlates with compensatory modulation of PV + interneuron function in DS (Nakakubo et al., 2023; Figure 2). The synthetic neuroactive steroid SGE-516, targeting both synaptic and extrasynaptic GABAA receptors, boosts 6-week survival to 100% in DS mice via a benzodiazepine-independent mechanism, highlighting broad GABAergic system modulation for refractory epilepsies (Hawkins et al., 2017; Figure 2).

Upstream regulatory strategies expand therapeutic horizons by reshaping GABAergic microenvironments: E2730, a novel GAT1 inhibitor that selectively inhibits GAT1-mediated GABA uptake, elevates extracellular GABA selectively under hyperactive synaptic conditions, minimizing basal GABA disruption and addressing the neurotoxicity of conventional antiseizure medications (ASMs; Fukushima et al., 2023; Figure 2). ABHD6 inhibition enhances tonic GABAA receptor currents in dentate granule cells without affecting synaptic transmission, an effect abrogated by GABAA receptor antagonists, revealing a unique “extrasynaptic inhibition potentiation” mechanism (Westenbroek et al., 2023).

The GluN2A-NMDA receptor modulator GNE-0723 represents a trans-level intervention from ion channels to neural oscillations: by enhancing synaptic NMDA currents in both excitatory and inhibitory neurons, it corrects abnormal low-frequency oscillations (12–20 Hz) and improves spatial memory in DS models. This links to restored synchrony of inhibitory interneurons, validating the “oscillatory dysregulation-circuit remodeling” hypothesis (Hanson et al., 2020). These strategies, targeting both direct *SCN1A* deficits and systemic excitatory-inhibitory imbalance, mark a shift from “symptom control” to “network homeostasis restoration” in DS therapy.

While ion channel-targeted therapies have enhanced the precision and safety of DS treatment through subtype-specific modulation and multi-target synergy, their clinical translation faces challenges including blood–brain barrier penetration efficiency, long-term neural adaptation, and cross-species efficacy variability. Future efforts may leverage AI-assisted drug design to optimize molecular structures and combine gene delivery technologies for cell-type-specific expression, propelling the field from single-channel modulation to comprehensive brain network reconstruction.

Selective inhibition of GAT1-mediated GABA reuptake enhances extracellular GABA concentrations, potentiating tonic inhibition. GS967, a selective Nav1.6 channel blocker, reduces Nav1.6 protein levels in excitatory pyramidal neurons, effectively suppressing hippocampal hyperexcitability. SGE-516 and KRM-II-81 prolong IPSC decay, preferentially enhancing postsynaptic inhibition to elevate seizure thresholds and ameliorate anxiety-like behaviors. The novel GAT1 inhibitor E2730 selectively elevates extracellular GABA under conditions of high synaptic activity while minimizing interference with basal GABA levels, addressing the neurotoxicity limitations of conventional antiepileptic drugs.

### 5.2 Neurotransmitter and neurotrophic factor regulation: multimodal interventions from receptor targeting to network remodeling

Serotonin (5-hydroxytryptamine, 5-HT)-based therapeutic strategies for epilepsy have gained traction in DS due to their dual potential to mitigate seizures and reduce epilepsy-related risks (such as sudden unexpected death in epilepsy, SUDEP). This section focuses on 5-hydroxytryptophan (5-HTP) supplementation, a strategy validated in preclinical models to enhance brain serotonin levels and modulate epileptogenic networks (Feng and Faingold, 2017; Gartside et al., 1992).

In DS context, serotonin dysregulation may contribute to disease pathophysiology via two key pathways:

Respiratory and cardiac homeostasis: Polymorphisms in *TPH2* (the rate-limiting enzyme for serotonin synthesis) reduce enzyme activity and brain serotonin levels in DBA/1 mice, increasing susceptibility to seizure-induced respiratory arrest (S-IRA; Zhang et al., 2004; Kulikov et al., 2005; Osipova et al., 2010). 5-HTP bypasses TPH2 to elevate serotonin levels (Gartside et al., 1992), reducing S-IRA incidence in sound- and PTZ-induced seizure models (Feng and Faingold, 2017). Specifically, acute 5-HTP (125–150 mg/kg) or repeated 100 mg/kg administration decreases S-IRA by 50–70% (Feng and Faingold, 2017), mirroring effects of selective serotonin reuptake inhibitors (SSRIs) in the same model (Faingold et al., 2011; Faingold and Randall, 2013).

Network excitability modulation: Serotonergic projections to the thalamus and cortex (via 5HT1A/2A receptors) dampen hyperexcitability in DS-relevant circuits. In DBA/1 mice, 5-HTP enhances potassium channel activity in cardiac myocytes (Faingold et al., 2011) and stabilizes respiratory neuron firing via calcium-dependent mechanisms (Zeng et al., 2015), mechanistically linking serotonin to both seizure control and vital sign regulation.

These findings highlight 5-HTP as a promising adjuvant therapy for DS, particularly for managing SUDEP risk and reducing seizure-induced respiratory compromise. Clinical trials evaluating 5-HTP in DS are warranted to translate these preclinical insights (Feng and Faingold, 2017).

**Figure 3:**
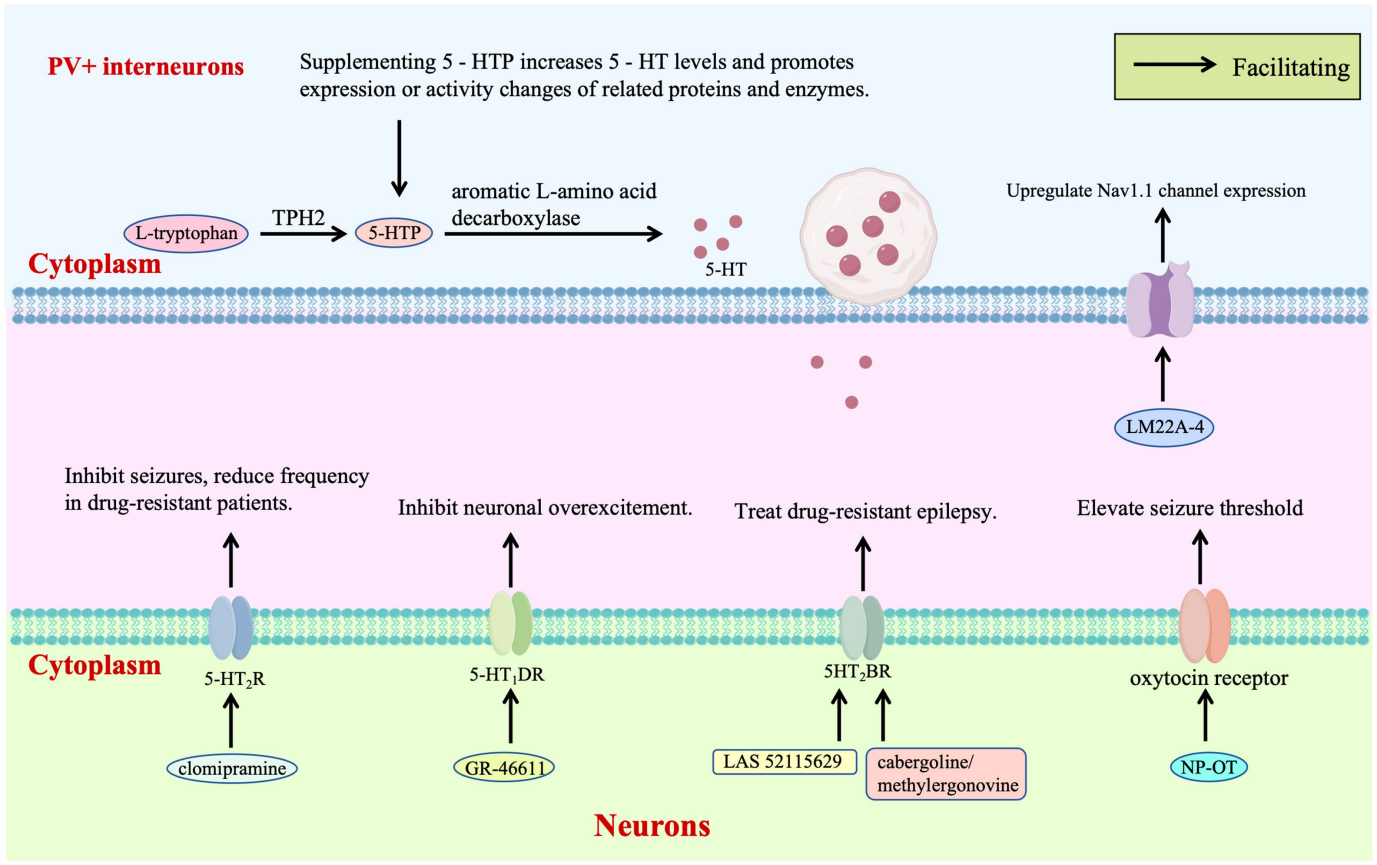
Regulatory targets modulate neurotransmitters and neurotrophic factors.

Receptor-targeted therapy of the 5-HT system provides a critical pathway for regulating the excitatory-inhibitory balance in DS. Zebrafish models and clinical observations have shown that clomipramine and 5-HT modulators such as trazodone and lorcaserin inhibit epileptic activity by acting on 5-HT₂A/B/C receptors, reducing seizure frequency in drug-resistant patients and clinically verifying the therapeutic potential of 5-HT₂R agonists for DS for the first time (Griffin et al., 2017; Figure 3). Further studies have demonstrated that the selective 5-HT₁D receptor agonist GR-46611 activates 5-HT₁D receptors in brain regions such as the thalamus, inhibiting excessive excitation of the forebrain network without interfering with thermoregulation. This intervention increases the survival rate of *SCN1A*+/− mice during heat-induced seizures from 36 to 89%, highlighting the targeted advantage of this receptor subtype in thermosensitive epilepsy (Hatini and Commons, 2020; Figure 3).

Novel 5HT2BR agonist LAS 52115629 stabilizes receptor conformation through strong hydrogen bonding with ASP135, exhibiting lower toxicity than existing drugs. Molecular dynamics reveal activation of G-protein signaling via conserved motifs, presenting a non-Nav1.1-targeted approach for drug-resistant cases (Chauhan et al., 2023; Figure 3). Virtual screening identifies FDA-approved cabergoline and methylergonovine as potent 5HT2BR agonists, inducing helical conformational changes through interactions with conserved residues and confirming non-mutagenic properties, which supports repurposing these agents to modulate 5HT2BR-mediated excitability (Chauhan et al., 2023; Figure 3).

Neurotrophic factor therapy introduces a new dimension by reshaping inhibitory neuron function: intranasal nanoparticle-encapsulated oxytocin (NP-OT) elevates seizure thresholds and restores social behavior in *Scn1a+/−* mice via oxytocin receptor activation, offering a non-invasive neuropeptide-based strategy without neurotoxicity (Wong et al., 2021; Figure 3). Early intervention with TrkB partial agonist LM22A-4 increases parvalbumin-positive inhibitory synapses around cortical pyramidal neurons, upregulates Nav1.1 expression in interneurons, and reduces spontaneous seizures from 65 to 21%, demonstrating neurotrophic modulation of inhibitory synaptic plasticity (Gu et al., 2022; Figure 3).

The AKT1 signaling pathway uncovers fine-grained regulation of neuronal excitability: AKT1 phosphorylates the intracellular loop of Nav1.1, shifting activation voltage by +10 mV, accelerating slow inactivation, and reducing peak sodium currents by 30–50% independent of surface expression. Opposite effects of SC79 and triciribine indicate AKT1 modulates channel gating to influence inhibitory interneuron firing, proposing AKT1 as a novel therapeutic target (Arribas-Blázquez et al., 2021).

While multi-dimensional interventions targeting neurotransmitter systems (5-HT receptor subtype modulation, GABAergic microenvironment remodeling) and neurotrophic factors (oxytocin, TrkB pathway) have provided cross-target strategies for DS, their clinical implementation is hindered by off-target effects from receptor functional crosstalk, insufficient tissue specificity in neurotrophic factor delivery, and developmental stage sensitivity in signaling pathway regulation (e.g., *AKT1*). Future research may leverage cryo-electron microscopy to resolve atomic-level receptor-ligand interactions, develop non-peptide small molecules with enhanced blood–brain barrier permeability, and establish individualized neural plasticity models through longitudinal multi-omics tracking, advancing the field from single-target modulation to systematic restoration of neural circuit homeostasis.

The 5-HT initiates with L-tryptophan, which is converted to 5-HTP by TPH —the rate-limiting enzyme in this pathway—and subsequently to 5-HT by aromatic L-amino acid decarboxylase. *TPH2* polymorphisms associated with reduced enzymatic activity may lead to 5-HT deficiency, increasing susceptibility to S-IRA. Supplementation with 5-HTP circumvents the *TPH2* bottleneck, directly elevating brain 5-HT levels to enhance ion channel protein function in respiratory neurons—thereby maintaining respiratory rhythm—and promote expression of potassium/calcium channels in cardiomyocytes to stabilize cardiac electrical activity. Early intervention with the TrkB partial agonist LM22A-4 enhances parvalbumin-positive inhibitory synapses surrounding cortical pyramidal neurons and upregulates Nav1.1 expression in interneurons, reinforcing inhibitory tone. Clinically, 5-HT modulators such as trazodone, lorcaserin, and clomipramine suppress epileptiform activity by targeting 5-HT2A/B/C R, reducing seizure frequency in drug-resistant patients. The selective 5-HT1D receptor agonist GR-46611 dampens forebrain network hyperexcitability by activating 5-HT₁D receptors in brain regions including the thalamus. Mechanistically, the novel 5HT2BR agonist LAS 52115629 stabilizes receptor conformation through a strong hydrogen bond with Asp135, demonstrating reduced toxicity compared to existing agents. Potent 5HT2BR agonists like cabergoline and methylergometrine induce helical conformational changes via interactions with conserved residues, fine-tuning 5HT2BR-mediated excitability. Intranasal oxytocin encapsulated in NP-OT elevates seizure thresholds in DS mice by activating oxytocin receptors, representing a non-invasive strategy for modulating neuroexcitability.

### 5.3 Genetic therapies

#### 5.3.1 Antisense oligonucleotide (ASO) targeted therapy: from gene expression correction to multi-target interventions

Antisense oligonucleotides (ASOs) are precision gene regulatory tools that, via base complementary pairing, target cellular RNA to achieve high-specificity modulation of key post-transcriptional processes. They offer an innovative strategy for targeting proteins traditionally considered “undruggable” and for developing personalized therapies based on patient-specific mutations (Kim et al., 2019; Carroll et al., 2011). The druggability of ASOs critically depends on chemical modification strategies (such as 2’-MOE for enhanced stability and PS backbone for resistance to degradation) to optimize their properties (Monia et al., 1993; McKay et al., 1999; Khvorova and Watts, 2017; Shen et al., 2019).

**Figure 4:**
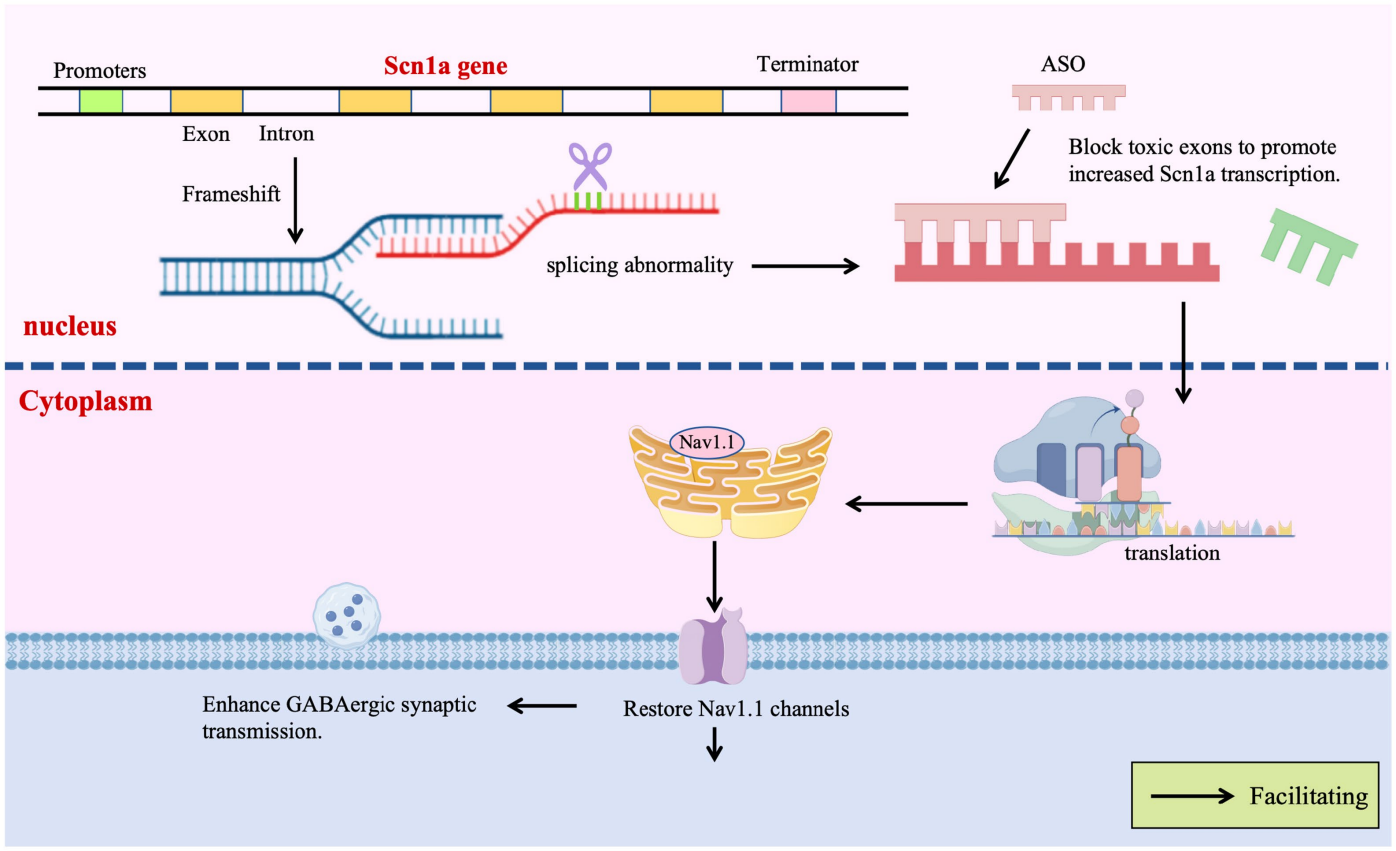
Gene therapy targets.

Given the potential of ASOs to precisely modify gene expression by regulating pre-mRNA splicing or stability, their application in DS therapy is a current research focus. Targeting the *SCN1A* gene, efforts are predominantly directed towards developing ASO-based strategies capable of upregulating *SCN1A* expression or restoring its function. ASO therapies offer revolutionary strategies for DS by precisely regulating gene expression, transcending traditional symptomatic treatments. A prime example is the Targeted Augmentation of Nuclear Gene Output (TANGO) technology, which exemplifies the power of splicing-based repair: Targeting the core defect of SCN1A splicing aberrations, TANGO technology achieves molecular-level repair: ASO-84 (a surrogate for STK-001) prevents inclusion of poison exon 20 N, increasing SCN1A transcripts 1.5-fold and restoring Nav1.1 protein to wild-type levels. This rescues sodium current density in PV + inhibitory interneurons and enhances GABAergic synaptic transmission, improving survival to 92% in DS mice with sustained efficacy into adulthood (Yuan et al., 2024). The clinical candidate STK-001, via TANGO, systemically enhances nuclear gene output to restore Nav1.1 levels to >80% of wild-type, improving electrophysiological properties and network connectivity of inhibitory neurons, laying critical preclinical groundwork for disease-modifying therapies (Wengert et al., 2022). The use of targeted augmentation of nuclear gene output technology that modulates naturally occurring, nonproductive splicing events to increase target gene and protein expression via antisense oligonucleotide has been shown to rescue the normal phenotype and markedly reduce epilepsy in mouse models of DS through an increase in Nav1.1 expression and a rescue of synaptic inhibition (Han et al., 2020; Yuan et al., 2024). The developed ASO-22 targets newly identified poison exons 1 N and 22 N, blocking splicing factor binding to restore full-length transcripts, achieving a survival rate of 97% in neonatal mice after early intervention, which provides a time-sensitive strategy for severe early-life phenotypes (Han et al., 2020; Tang et al., 2025; Figure 4).

The scope of ASO therapy is expanding from single-gene correction to multi-target regulation: *tau*-targeting ASOs reduce excitatory neuron hyperexcitability by inhibiting PI3K-AKT–mTOR pathway overactivation, decreasing seizure frequency, improving autism-like behaviors, and preventing megalencephaly in DS mice, demonstrating potential for correcting neurodevelopmental abnormalities (Shao et al., 2022; Figure 4). *SCN8A*-targeting ASOs degrade Nav1.6 transcripts to selectively reduce sodium currents in excitatory neurons, extending median lifespan from 3 weeks to over 5 months in DS models without apparent side effects, revealing a cross-sodium channel therapeutic strategy (Lenk et al., 2020; Figure 4).

Long non-coding RNA modulation shows cross-species promise: AntagoNATs targeting *SCN1ANAT* upregulate *SCN1A* expression in mice and non-human primates, reducing spontaneous seizures and increasing thermal seizure thresholds. Safety in primates provides translational evidence for RNA interference-based gene expression regulation (Hsiao et al., 2016).

These strategies, extending beyond *SCN1A* splicing to target *tau*, *SCN8A*, and other pathways, highlight the diverse potential of ASOs in correcting gene expression, remodeling neural circuits, and regulating cellular signaling. The evolution from single-gene repair to multi-target interventions marks a shift in DS treatment toward precision and personalization based on molecular pathogenesis (Table 3).

**Table 3:** Summary of key genetic therapeutic strategies for DS.

Attribute	ASO	AAV-Mediated Gene Supplementation	CRISPR/dCas9 Transcriptional Activation (e.g., VPR)	Gene Editing / Repair
Primary Mechanism	Modulates pre-mRNA splicing (e.g., blocks inclusion of poison exons); increases full-length functional transcript.	Delivers functional *SCN1A* cDNA or engineered transcription factors to enhance *SCN1A* expression.	Enhances endogenous *SCN1A* transcription using dCas9-VPR fusion protein guided by gRNAs.	Corrects pathogenic mutations or conditionally activates gene expression (e.g., Gabra2 repair, conditional *SCN1A* activation).
Target Gene/Pathway	*SCN1A* (poison exons), *TAU*, *SCN8A*	*SCN1A*	*SCN1A* promoter region	*GABRA2, SCN1A*
Key Delivery Method	Intrathecal injection	Intravenous (IV) or Intracerebroventricular (ICV) injection	AAV (IV, ICV)	Likely AAV
Key Advantages	High specificity; splice correction potential; transient & reversible effect.	Potential for long-term expression; efficient CNS transduction; cell-type specific promoters available.	Precise upregulation of endogenous gene; DNA cleavage-free (safer); potential for cell-type specificity.	Potential for permanent correction; precise mutation repair.
Key Challenges/Limitations	Requires repeated dosing; limited BBB penetration (needs IT delivery); potential off-target effects; high cost.	Limited packaging capacity (large *SCN1A* cDNA); immunogenicity; potential insertional mutagenesis risk (low); high cost; optimal timing unclear.	Large construct size (packaging challenge); potential off-target epigenetic effects; immunogenicity; long-term safety/regulation unknown.	Editing efficiency; delivery challenges; off-target editing risk; safety concerns with editing tools; applicability to diverse mutations.
Representative Examples	TANGO technology (e.g., ASO-84, STK-001); ASO-22 (targeting ex1N/22 N); *Tau*-ASO; *Scn8a*-ASO	AAV9-*SCN1A* (pan-neuronal); AAV9-eTF*SCN1A* (activates endogenous promoter); Split-intein system (DLX2.0-*SCN1A*); HC-AdVs	dCas9-VPR + gRNAs (e.g., sg1P targeting proximal promoter)	*Gabra2* gene repair; Conditional *SCN1A* reactivation

STK-001 is an ASO-based therapy currently in clinical trials for DS. AAV = Adeno-Associated Virus; CNS = Central Nervous System; gRNA = guide RNA; IT = Intrathecal; IV = Intravenous; ICV = Intracerebroventricular. Detailed descriptions of mechanisms, preclinical results, and specific references for each strategy are provided in subsections 5.3.1 to 5.3.4.

#### 5.3.2 Adenovirus-mediated gene supplementation: from vector innovation to circuit-specific repair in DS

In the gene therapy for DS, the adeno-associated virus AAV9 is engineered to deliver the transcription factor *eTFSCN1A*, addressing the challenge that the *SCN1A* gene (encoding the voltage-gated sodium channel Na𝙑.1) exceeds the payload capacity of AAVs (>4.7 kb). This strategy employs *eTFSCN1A* to activate the endogenous *SCN1A* promoter, combined with GABAergic interneuron-specific regulatory elements, thereby achieving precise upregulation of sodium channel expression in inhibitory neurons and restoring neural circuit balance, restoring action potential firing and remodeling GABAergic neurotransmission without directly replacing mutant genes. This process compensates for voltage-gated sodium channel dysfunction, correcting the excitatory-inhibitory imbalance in neuronal networks. In *Scn1a+/−* mouse models, this approach significantly improves survival and reduces seizures, with efficacy closely linked to dose-dependent restoration of inhibitory synaptic transmission efficiency (Yuan et al., 2024; Figure 4).

To address GABAergic neuron sodium channel deficits from *SCN1A* heterozygous loss-of-function in DS, the AAV9-*eTFSCN1A* vector uses an engineered transcription factor with GABAergic-specific elements to selectively activate the endogenous *SCN1A* promoter, upregulating Nav1.1 expression. This strategy reduces seizures and prolongs survival in *Scn1a+/−* mice, with non-human primates showing widespread brain expression and safety. Overcoming AAV packaging limits, this endogenous regulation approach highlights subtype-specific therapeutic potential for pathogenic neuronal populations (Tanenhaus et al., 2022; Patel et al., 2024).

Adenovirus-based gene supplementation therapy drives the expression of the sodium channel auxiliary subunit NaVβ1 via the *Gad-1* promoter, specifically targeting GABAergic interneurons to restore Nav1.1 function by enhancing the membrane localization of residual channels, thereby offering a therapeutic approach for *SCN1A* haploinsufficiency in DS. This strategy leverages the precise cell-type specificity of the Gad-1 promoter to achieve sustained neuronal expression while minimizing off-target risks associated with non-specific promoters. Notably, preclinical studies have shown that AAV-*NaVβ1* treatment reduces mortality by 40% in female *Scn1a+/−* mice and ameliorates hyperactivity/anxiety-like behaviors in males, with this sex-dependent efficacy linked to baseline differences in GABAergic neuronal activity between sexes. These findings highlight the neuroregulatory microenvironment as a key modulator of therapeutic response, emphasizing the multidimensional utility of auxiliary protein-mediated Nav1.1 compensation in addressing *SCN1A* haploinsufficiency (Yuan et al., 2024; Niibori et al., 2020). Furthermore, the 4.7 kb single-stranded packaging capacity limit of AAV vectors precludes direct delivery of the *SCN1A* gene encoding Nav1.1 (approximately 6 kb), as DNA fragments exceeding this capacity lead to vector packaging failure or functional loss. Additionally, sole expression of Nav1.1 may fail to properly fold or localize due to the lack of auxiliary subunit regulation. In contrast, the NaVβ1 cDNA (approximately 1.2 kb) allows for controllable total length when combined with promoters and regulatory elements. It achieves therapeutic effects by enhancing Nav1.1 membrane localization, regulating channel kinetics, and compensating for downregulated endogenous NaVβ1, demonstrating the breakthrough of the “auxiliary subunit substitution + tissue-specific promoter” strategy against AAV capacity constraints and its functional compensation advantages (Yuan et al., 2024).

The split-intein dual-vector system (*DLX2.0*-*SCN1A*) represents a milestone in targeting precision, achieving interneuron-specific *SCN1A* expression to reduce postnatal mortality by >50% and seizure frequency by 60% in a dose-dependent manner. By avoiding preweaning lethality associated with pan-neuronal expression, this strategy validates cell-type specificity as a cornerstone for safe gene therapy design (Mich et al., 2025).

High-capacity adenoviral vectors (HC-AdVs) with the hybrid *DP3V* promoter enable efficient *SCN1A* expression in GABAergic neurons, controlling heat-induced seizures and improving survival in DS models. Adolescent intervention studies show that Nav1.1 elevation in basal ganglia, cerebellum, and prefrontal cortex enhances motor coordination and exploratory behavior, validating functional compensation for haploinsufficiency despite unchanged spatial learning (Ricobaraza et al., 2023; Mora-Jimenez et al., 2021).

Deletion of the non-canonical *SCN1A* 1b regulatory region, which reduces brain-wide Nav1.1 by 57–76% in cortex, highlights the need to incorporate non-coding regulatory elements into vector design. This expands gene therapy targets beyond coding sequences, providing a rationale for optimizing promoter-enhancer combinations to improve expression efficiency (Haigh et al., 2021).

Adenovirus-mediated gene supplementation provides precise repair for *SCN1A* dysfunction in DS through neuron subtype-specific delivery and endogenous gene regulation. Current strategies demonstrate inhibitory circuit remodeling and cross-developmental therapeutic potential in animal models, yet face translation challenges including AAV packaging constraints, long-term efficacy monitoring, and sex-related response variability. Future research may focus on integrating non-coding regulatory elements with multi-promoter systems and combining with ion channel-targeted drugs to establish synergistic intervention, advancing gene therapy from single-molecule compensation to systematic restoration of neural circuit function (Table 3).

#### 5.3.3 CRISPR/dCas9-VPR activates the *SCN1A* promoter, enhancing neuron-specific Nav1.1 expression

The CRISPR/dCas9-VPR system utilizes guide RNA-directed targeting of catalytically dead Cas9 (dCas9) fused to the transcriptional activator VPR to specifically upregulate endogenous *SCN1A* expression from its native promoter. By employing inhibitory neuron-specific promoters (e.g., *mDlx5/6*), ectopic expression in excitatory neurons is prevented, mitigating potential neurotoxicity risks. This DNA cleavage-free approach substantially reduces off-target effects and genomic instability while demonstrating therapeutic efficacy in DS models: a single administration rescues action potential firing in GABAergic interneurons and elevates seizure thresholds, directly countering *SCN1A* haploinsufficiency (Guan et al., 2022; Ricci and Colasante, 2021).

The CRISPR/dCas9-VPR system recruits transcriptional activators to the endogenous *SCN1A* promoter via four gRNAs (including sgRNA sg1P targeting the proximal promoter), specifically enhancing Nav1.1 expression in inhibitory interneurons. *In vivo* AAV delivery restores neuronal firing patterns, elevates seizure thresholds, reduces hippocampal hyperactivity, and improves motor/social behaviors in DS mice—demonstrating that endogenous gene activation repairs neural circuits without exogenous DNA integration (Gu et al., 2022; Colasante et al., 2020; Figure 4).

A CRISPR/dCas9-VPR system with four upstream promoter-targeting gRNAs significantly boosts Nav1.1 levels in inhibitory interneurons. AAV-based treatment elevates thermal seizure thresholds, reduces hippocampal spikes, and improves motor and social behaviors in DS mice. By enhancing endogenous promoter activity through a transcriptional activator complex, this neuron-specific strategy provides a novel path for precision gene therapy (Yamagata et al., 2020).

The CRISPR/dCas9-VPR system has demonstrated precision in regulating *SCN1A* expression in DS models, yet its clinical translation hinges on optimizing delivery systems and ensuring long-term safety. Future research could explore non-viral delivery platforms to reduce immunogenicity and develop molecular tools for dynamic monitoring of gene expression to enable real-time assessment of therapeutic efficacy. Additionally, integrating epigenetic editing technologies may enhance the durability of promoter activation, offering more stable intervention strategies for monogenic neurodevelopmental disorders (Table 3).

#### 5.3.4 Single-nucleotide editing repairs the Gabra2 gene or conditionally activates *SCN1A*, reversing established pathological phenotypes

Research has found that in a mouse model of DS, single-nucleotide editing and repair of the Gabra2 gene can increase its transcriptional and protein expression levels, as well as the number of α2-containing GABAA receptors in hippocampal synapses. This repair significantly improves the epileptic phenotype of mice, increasing survival rates and reducing the frequency and severity of seizures. This indicates that enhancing the function of GABRA2 holds promise as a novel therapeutic strategy for DS, offering potential drug intervention targets for clinical treatment (Hawkins et al., 2021).

Using a conditional *SCN1A* knock-in model, this study showed that reactivating *SCN1A* at symptom onset (P30) fully eliminated seizures, improved hyperactivity and social deficits, and normalized hippocampal interneuron function. Restoring Nav1.1 in adult mice (P90) also controlled chronic seizures and reversed astrogliosis-relat*Scn1a*ed gene changes. These findings demonstrate that reconstituting *SCN1A* activity can reverse established Dravet phenotypes, even after symptom emergence, offering a proof-of-concept for post-onset gene repair strategies (Valassina et al., 2022).

While gene editing to repair GABRA2 or activate has shown promise in improving neurological function in DS models, challenges remain in achieving precise delivery and ensuring long-term safety. Future efforts may focus on optimizing neuronal targeting of AAV vectors, enhancing repair efficiency with novel base-editing technologies, and developing non-invasive monitoring methods to assess therapeutic efficacy. If translational hurdles can be overcome, such endogenous gene regulation strategies may offer disease-modifying therapies for neurodevelopmental disorders (Table 3).

ASO therapies targeting *SCN1A* prevent pathogenic exon inclusion during pre-mRNA splicing, thereby enhancing *SCN1A* transcript levels and restoring functional Nav1.1 sodium channel expression to potentiate GABAergic synaptic transmission in inhibitory interneurons. Concurrently, *tau*-targeting ASOs mitigate hyperexcitability in excitatory neurons by inhibiting excessive PI3K-AKT–*mTOR* pathway activation, reducing seizure frequency in DS. *Scn8a*-directed ASOs selectively degrade Nav1.6 transcripts, dampening sodium currents in excitatory neurons to alleviate network hyperexcitability. Gene therapy approaches, including AAV-mediated enhancement of the endogenous *SCN1A* promoter and CRISPR/dCas9-driven transcriptional activation, selectively augment Nav1.1 sodium channel expression in inhibitory interneurons. These interventions normalize action potential firing, restore GABAergic neurotransmission, and re-establish physiological membrane potentials, addressing the core excitatory-inhibitory imbalance underlying DS pathogenesis.

### 5.4 Signaling pathway and metabolic interventions: multidimensional therapeutic exploration from molecular targets to network regulation

In DS, mTOR signaling hyperactivation disrupts neuronal excitability by impairing sodium channel trafficking and enhancing glutamate release. mTOR inhibitors (e.g., everolimus) reduce hippocampal hyperexcitability by 40% and elevate seizure thresholds in models, supporting their repurposing for drug-resistant cases, particularly those with cortical dysplasia comorbidity (Tsai et al., 2025). *SCN1A* deficiency induces mitochondrial dysfunction and redox imbalance. Dimercaprol increases glutathione by 50–70%, suppressing hyperexcitability as a potential adjuvant to standard antiseizure medications (Sri Hari et al., 2023).

For proteostasis defects, zonisamide enhances degradation of misfolded GABAA receptor γ2 subunits, restoring receptor density by 2.5-fold in models of *GABRG2*-variant DS, offering a precision approach for genetically defined subpopulations (Poliquin et al., 2024). Microglial modulation may mitigate circuit hyperexcitability but requires brain-penetrant agents for clinical translation (Brenet et al., 2024).

Critical next steps for clinical implementation include:

1. Phase II trials of mTOR inhibitors in drug-resistant DS.
2. Biomarker development (e.g., hippocampal lactate/glutamate ratios) to identify redox modulator responders.
3. Combinatorial testing of pathway-specific agents (e.g., everolimus + dimercaprol).

However, mTOR inhibitor trials must address pediatric-specific immunosuppression risks (e.g., ≥20% infection rates in oncology), demanding real-world safety monitoring protocols. Biomarker development faces technical hurdles in translating hippocampal metabolite ratios to clinically feasible CSF/blood assays. Combinatorial strategies risk unforeseen pharmacodynamic interactions—exemplified by redox modulators potentially exacerbating zonisamide-induced carbonic anhydrase inhibition.

### 5.5 Novel technologies driving precision medicine: electrophysiological typing and drug repurposing in interdisciplinary therapies

Interdisciplinary approaches are revolutionizing DS management. The *SCN1A* p. R1636Q gain-of-function mutation, associated with neonatal epileptic encephalopathy, exhibits distinct electrophysiological features: a significantly left-shifted activation voltage (−27.2 mV vs. −21.2 mV in wild-type), fourfold slower inactivation kinetics, and a fivefold increase in persistent sodium current (22.6% vs. 4.7% in wild-type). This gain-of-function phenotype, distinct from classical haploinsufficiency, confers sensitivity to the voltage-gated sodium channel blocker oxcarbazepine, with reduced seizure frequency in affected patients, highlighting the clinical utility of electrophysiological profiling for personalized treatment (Clatot et al., 2023).

Computational biology unlocks new avenues for drug-resistant DS: virtual screening and molecular dynamics identify FDA-approved cabergoline (−53.44 kcal/mol binding energy) and methylergonovine (−40.42 kcal/mol) as potent 5HT2BR agonists. These drugs induce a 2.48–3.07 Å conformational change in the receptor’s helical domain by binding conserved residues ASP135 and LEU209, enhancing G-protein coupling efficiency. With non-mutagenic properties confirmed by ADMET analysis, this structure-based repurposing strategy bypasses lengthy drug development pipelines, offering a rapid therapeutic solution for resistant cases and exemplifying interdisciplinary innovation in rare disease therapy (Chauhan et al., 2023).

Interdisciplinary approaches such as electrophysiological typing and drug repurposing are carving out new pathways for precision therapy in DS, with electrophysiology-informed personalized medication and structure-based repurposing strategies showing promise in specific mutational subtypes. Current challenges include the lack of standardized clinical protocols for electrophysiological phenotyping and the need for large-scale cohort studies to validate the target specificity and long-term safety of repurposed drugs. Future investigations integrating single-cell electrophysiological profiling with multi-omics datasets could establish more precise molecular-electrophysiological correlation models, facilitating efficient translation of interdisciplinary technologies from mechanistic discovery to clinical application.

## 6 Conclusion

DS, a severe neurodevelopmental disorder dominated by *SCN1A* dysfunction, exhibits profound mechanistic complexity: *SCN1A* mutations disrupt Nav1.1 expression in GABAergic interneurons through transcriptional dysregulation, pre-mRNA splicing defects, and voltage-gated sodium channel dysfunction, leading to impaired inhibitory synaptic transmission and brainwide excitatory-inhibitory imbalance. Polygenic variants (e.g., *DEPDC5, CHD2*), astrocytic calcium signaling aberrations, and mitochondrial metabolic perturbations synergistically exacerbate network hyperexcitability, underscoring the disease’s multifactorial pathogenesis.

Diagnostic advancements have established a lifecycle-precise framework by integrating early febrile seizure phenotypes, comprehensive *SCN1A* sequencing (including deep intronic variants), and multimodal assessments (e.g., γ-band EEG power analysis, hippocampal volumetry), significantly improving diagnostic efficiency and etiological clarity. Therapeutic innovations pivot from empirical seizure control to mechanism-targeted strategies: antisense oligonucleotides restore *SCN1A* transcript integrity by blocking pathogenic exon inclusion; AAV/CRISPR-dCas9 technologies selectively enhance Nav1.1 expression in inhibitory neurons to rebalance GABAergic transmission; and novel ion channel modulators (e.g., Nav1.1-selective agonist Hm1a) and serotonin system-targeted agents regulate neuronal electrophysiology and neurotransmitter homeostasis, offering new options for drug-resistant cases.

These findings not only delineate the cascading pathogenesis from molecular defects to network dysfunction but also validate the therapeutic potential of “inhibitory neuron-targeted repair” through interdisciplinary integration of structural biology, electrophysiological phenomics, and gene editing. Notably, the correlation between *SCN1A* variant location (coding vs. non-coding) and pathogenic effects, the modulating role of polygenic backgrounds in phenotypic diversity, and the active participation of neuroglial cells in epileptogenic network formation provide critical insights into DS clinical heterogeneity. The established molecular diagnostic-therapeutic framework not only paves the way for precision management of DS but also serves as a paradigmatic approach for dissecting complex mechanisms of monogenic neurodevelopmental disorders, offering transferable methodologies to related fields.

## 7 Outlook

The precision management of DS faces critical challenges and opportunities, with future research prioritizing the following frontiers:

1. Retinal-Brain Glial Dynamics as a Diagnostic Window: Building on the potential of non-invasive biomarkers like retinal imaging (e.g., OCT) highlighted for capturing glial activity, future studies should decipher the bidirectional signaling between retinal and brain glial cells (astrocytes, microglia; Salazar et al., 2023). This research aims to establish validated retinal signatures as surrogate markers for central nervous system pathology, ultimately paving the way for OCT-based non-invasive monitoring tools for early diagnosis and treatment response assessment in DS.
2. Targeting Novel Lipid Signaling Hubs in Seizure Pathogenesis: Leveraging metabolomic insights into altered lipid mediators, including specific monoacylglycerols, during seizure states, future efforts must elucidate the precise pathogenic roles of molecules like 2-LG and 1-LG in network hyperexcitability (Bahceci et al., 2023). This involves mapping their synthesis, degradation pathways, and receptor signaling, with the goal of developing novel therapeutic strategies that specifically modulate these lipid-signaling cascades to overcome limitations of current metabolic interventions for drug-resistant epilepsy.
3. Precision intervention during the critical developmental window: The ENVISION study underscores that language delays in *SCN1A* + DS emerge irreversibly after age 2 years, independent of seizure burden (Fine, 2024), emphasizing the neuroplastic critical period in infancy. Integrating single-cell sequencing and electrophysiology, future strategies should prioritize early gene therapies (e.g., AAV/CRISPR) to enhance Nav1.1 expression in GABAergic interneurons before age 2, combined with neurorehabilitation to optimize synaptic plasticity, aiming to disrupt pathological cascades prior to developmental stagnation.
4. Neuroimmune modulation and microglial function targeting: DS models exhibit pro-inflammatory microglia with phagocytic deficits due to Nav1.1 loss, leading to excessive immature synapses in the dentate gyrus (Chen et al., 2024). This opens avenues for neuroimmune-targeted therapies: small molecules (e.g., cannabidiol) or genetic approaches could regulate microglial polarization and enhance synaptic pruning, while minimizing risks of immunosuppression. Defining region-specific effects and long-term safety remains essential.
5. Targeting compensatory ion channels for circuit repair: Reduced SK2 channel (*Kcnn2S*) expression in thalamic reticular neurons drives abnormal bursting in DS models, reversible by SK agonists (Isom, 2019). Beyond conventional GABAergic modulation, this highlights compensatory ion channels (SK2, Nav1.6) as viable targets. Repurposing existing drugs (e.g., chlorzoxazolone) or developing subtype-specific modulators could address late-stage neurocircuit abnormalities, requiring integration with electrophysiological phenotyping to refine therapeutic specificity.

In summary, future research must integrate multi-omics, single-cell technologies, and cross-organ pathology to decode DS from molecular defects to circuit dysfunction, driving a paradigm shift from symptomatic control to mechanistic correction.

## Glossary

DS: Dravet Syndrom
ASOs: Antisense oligonucleotides
AAV: Adeno-associated virus
SMEI: Severe myoclonic epilepsy of infancy
SUDEP: Sudden unexpected death in epilepsy
PV+: Parvalbumin-positive
AIS: Axon initial segments
PVINs PV+: fast-spiking interneurons
E-I: Excitation-inhibition
SIPSCs: Spontaneous inhibitory postsynaptic currents
BNST: Bed nucleus of the stria terminalis
ATP: Adenosine triphosphate
GABA γ: aminobutyric acid
FS+: Fever-induced generalized tonic–clonic seizures
SEP: Seizure-evoked potential
VUS: Variants of uncertain significance
CDSS: Clinical decision support systems
AEDs: Antiepileptic drugs
VPA: Valproic acid
CLB: Clobazam
CBD: Cannabidiol
FFA: Fenfluramine
ASMs: Antiseizure medications
IPSC: Inhibitory postsynaptic current
5-HTP: 5-hydroxytryptophan
TPH2: Tryptophan hydroxylase-2
S-IRA: Seizure-induced respiratory arrest
NP-OT: Nanoparticle-encapsulated oxytocin
TANGO: Targeted Augmentation of Nuclear Gene Output
HC-AdVs: High-capacity adenoviral vectors
CRISPR: Clustered regularly interspaced short palindromic repeats
spCas9: *Streptococcus pyogenes* Cas9
dCas9: Dead Cas9
CRISPRi: CRISPR interference
CRISPRa: CRISPR activation
DSBs: DNA double-strand breaks
gRNA: Guide RNA
DMP: Dimercaprol
GSH: Glutathione
OCT: Coherence tomography
5-HT: 5-hydroxytryptamine, serotonin

## References

[ref2] Almog Y. Fadila S. Brusel M. Mavashov A. Anderson K. Rubinstein M. (2021). Developmental alterations in firing properties of hippocampal CA1 inhibitory and excitatory neurons in a mouse model of Dravet syndrome. Neurobiol. Dis. 148:105209. doi: 10.1016/j.nbd.2020.105209, PMID: 33271326

[ref3] Alonso C. García-Culebras A. Satta V. Hernández-Fisac I. Sierra Á. Guimaré J. A.. (2025). Investigation in blood-brain barrier integrity and susceptibility to immune cell penetration in a mouse model of Dravet syndrome. Brain Behav. Immun. Health 44:100955. doi: 10.1016/j.bbih.2025.100955, PMID: 40028233 PMC11869101

[ref4] Anderson L. L. Hawkins N. A. Thompson C. H. Kearney J. A. George A. L. Jr. (2017). Unexpected efficacy of a novel Sodium Channel modulator in Dravet syndrome. Sci. Rep. 7:1682. doi: 10.1038/s41598-017-01851-9, PMID: 28490751 PMC5431801

[ref5] Anwar A. Saleem S. Patel U. K. Arumaithurai K. Malik P. (2019). Dravet Syndrome: An Overview. Cureus. 11:e5006. doi: 10.7759/cureus.5006, PMID: 31497436 PMC6713249

[ref6] Arribas-Blázquez M. Piniella D. Olivos-Oré L. A. Bartolomé-Martín D. Leite C. Giménez C.. (2021). Regulation of the voltage-dependent sodium channel NaV1.1 by AKT1. Neuropharmacology 197:108745. doi: 10.1016/j.neuropharm.2021.108745, PMID: 34375627

[ref7] Bahceci D. Anderson L. L. Kevin R. C. Doohan P. T. Arnold J. C. (2023). Hyperthermia-induced seizures enhance brain concentrations of the endocannabinoid-related Linoleoyl Glycerols in a Scn1a+/− mouse model of Dravet syndrome. Cannabis Cannabinoid Res. 8, 495–504. doi: 10.1089/can.2022.0145, PMID: 36269656

[ref8] Black J. A. Waxman S. G. (2013). Noncanonical roles of voltage-gated sodium channels. Neuron 80, 280–291. doi: 10.1016/j.neuron.2013.09.012, PMID: 24139034

[ref9] Bonvento G. Bolaños J. P. (2021). Astrocyte-neuron metabolic cooperation shapes brain activity. Cell Metab. 33, 1546–1564. doi: 10.1016/j.cmet.2021.07.006, PMID: 34348099

[ref10] Brandt C. Lahr D. May T. W. (2015). Cognitive adverse events of topiramate in patients with epilepsy and intellectual disability. Epilepsy Behav. 45, 261–264. doi: 10.1016/j.yebeh.2014.12.043, PMID: 25843340

[ref11] Brenet A. Somkhit J. Csaba Z. Ciura S. Kabashi E. Yanicostas C.. (2024). Microglia mitigate neuronal activation in a zebrafish model of Dravet syndrome. Cells 13:684. doi: 10.3390/cells13080684, PMID: 38667299 PMC11049242

[ref12] Bromfield E. B. Dworetzky B. A. Wyszynski D. F. Smith C. R. Baldwin E. J. Holmes L. B. (2008). Valproate teratogenicity and epilepsy syndrome. Epilepsia 49, 2122–2124. doi: 10.1111/j.1528-1167.2008.01696.x, PMID: 18557775

[ref13] Brunklaus A. Pérez-Palma E. Ghanty I. Xinge J. Brilstra E. Ceulemans B.. (2022). Development and validation of a prediction model for early diagnosis of *SCN1A*-related epilepsies. Neurology 98, e1163–e1174. doi: 10.1212/WNL.0000000000200028, PMID: 35074891 PMC8935441

[ref14] Bryson A. Reid C. Petrou S. (2023). Fundamental neurochemistry review: GABAA receptor neurotransmission and epilepsy: principles, disease mechanisms and pharmacotherapy. J. Neurochem. 165, 6–28. doi: 10.1111/jnc.15769, PMID: 36681890

[ref15] Cannon S. C. (2021). Epilepsy channelopathies go neddy: stabilizing NaV1.1 channels by neddylation. J. Clin. Invest. 131:e148370. doi: 10.1172/JCI148370, PMID: 33855971 PMC8262487

[ref16] Carroll J. B. Warby S. C. Southwell A. L. Doty C. N. Greenlee S. Skotte N.. (2011). Potent and selective antisense oligonucleotides targeting single-nucleotide polymorphisms in the Huntington disease gene / allele-specific silencing of mutant huntingtin. Mol. Ther. 19, 2178–2185. doi: 10.1038/mt.2011.201, PMID: 21971427 PMC3242664

[ref17] Carvill G. L. Engel K. L. Ramamurthy A. Cochran J. N. Roovers J. Stamberger H.. (2018). Aberrant inclusion of a poison exon causes Dravet syndrome and related SCN1A-associated genetic epilepsies. Am. J. Hum. Genet. 103, 1022–1029. doi: 10.1016/j.ajhg.2018.10.023, PMID: 30526861 PMC6288405

[ref18] Catterall W. A. (2010). Ion channel voltage sensors: structure, function, and pathophysiology. Neuron 67, 915–928. doi: 10.1016/j.neuron.2010.08.021, PMID: 20869590 PMC2950829

[ref19] Chauhan A. Sangwan N. Singh J. Prakash A. Medhi B. Avti P. K. (2023). Allosteric modulation of conserved motifs and helices in 5HT2BR: advances drug discovery and therapeutic approach towards drug resistant epilepsy. J. Biomol. Struct. Dyn. 41, 13113–13126. doi: 10.1080/07391102.2023.2178508, PMID: 36809314

[ref20] Chauhan A. Singh J. Sangwan N. Singh H. Prakash A. Medhi B.. (2023). Designing the 5HT2BR structure and its modulation as a therapeutic target for repurposing approach in drug-resistant epilepsy. Epilepsy Res. 194:107168. doi: 10.1016/j.eplepsyres.2023.107168, PMID: 37302343

[ref21] Chen I. C. Ho S. Y. Tsai C. W. Chen E. L. Liou H. H. (2024). Microglia-impaired phagocytosis contributes to the Epileptogenesis in a mouse model of Dravet syndrome. Int. J. Mol. Sci. 25:12721. Published 2024 Nov 27. doi: 10.3390/ijms252312721, PMID: 39684432 PMC11641197

[ref22] Chilcott E. Díaz J. A. Bertram C. Berti M. Karda R. (2022). Genetic therapeutic advancements for Dravet syndrome. Epilepsy Behav. 132:108741. doi: 10.1016/j.yebeh.2022.108741, PMID: 35653814

[ref23] Chow C. Y. Chin Y. K. Y. Ma L. Undheim E. A. B. Herzig V. King G. F. (2020). A selective NaV1.1 activator with potential for treatment of Dravet syndrome epilepsy. Biochem. Pharmacol. 181:113991. doi: 10.1016/j.bcp.2020.113991, PMID: 32335140

[ref24] Claes L. del-Favero J. Ceulemans B. Lagae L. van Broeckhoven C. de Jonghe P. (2001). De novo mutations in the sodium-channel gene SCN1A cause severe myoclonic epilepsy of infancy. Am. J. Hum. Genet. 68, 1327–1332. doi: 10.1086/320609, PMID: 11359211 PMC1226119

[ref25] Clatot J. Parthasarathy S. Cohen S. McKee J. L. Massey S. Somarowthu A.. (2023). SCN1A gain-of-function mutation causing an early onset epileptic encephalopathy. Epilepsia 64, 1318–1330. doi: 10.1111/epi.17444, PMID: 36287100 PMC10130239

[ref26] Colasante G. Lignani G. Brusco S. di Berardino C. Carpenter J. Giannelli S.. (2020). dCas9-based Scn1a gene activation restores inhibitory interneuron excitability and attenuates seizures in Dravet syndrome mice. Mol. Ther. 28, 235–253. doi: 10.1016/j.ymthe.2019.08.018, PMID: 31607539 PMC6952031

[ref27] de Lange I. M. Gunning B. Sonsma A. C. M. van Gemert L. van Kempen M. Verbeek N. E.. (2018). Influence of contraindicated medication use on cognitive outcome in Dravet syndrome and age at first afebrile seizure as a clinical predictor in SCN1A-related seizure phenotypes. Epilepsia 59, 1154–1165. doi: 10.1111/epi.14191, PMID: 29750338

[ref28] Debanne D. Mylonaki K. Musella M. L. Russier M. (2024). Voltage-gated ion channels in epilepsies: circuit dysfunctions and treatments. Trends Pharmacol. Sci. 45, 1018–1032. doi: 10.1016/j.tips.2024.09.004, PMID: 39406591

[ref29] Devinsky O. Cross J. H. Laux L. Marsh E. Miller I. Nabbout R.. (2017). Trial of Cannabidiol for drug-resistant seizures in the Dravet syndrome. N. Engl. J. Med. 376, 2011–2020. doi: 10.1056/NEJMoa1611618, PMID: 28538134

[ref30] Devinsky O. Nabbout R. Miller I. Laux L. Zolnowska M. Wright S.. (2019). Long-term cannabidiol treatment in patients with Dravet syndrome: an open-label extension trial. Epilepsia 60, 294–302. doi: 10.1111/epi.14628, PMID: 30582156 PMC7379690

[ref31] Du J. Vegh V. Reutens D. C. (2019). Small changes in synaptic gain lead to seizure-like activity in neuronal network at criticality. Sci. Rep. 9:1097. doi: 10.1038/s41598-018-37646-9, PMID: 30705357 PMC6355815

[ref32] Dyment D. A. Schock S. C. Deloughery K. Tran M. H. Ure K. Nutter L. M. J.. (2020). Electrophysiological alterations of pyramidal cells and interneurons of the CA1 region of the Hippocampus in a novel mouse model of Dravet syndrome. Genetics 215, 1055–1066. doi: 10.1534/genetics.120.303399, PMID: 32554600 PMC7404236

[ref33] Faingold C. L. Randall M. (2013). Effects of age, sex, and sertraline administration on seizure-induced respiratory arrest in the DBA/1 mouse model of sudden unexpected death in epilepsy (SUDEP). Epilepsy Behav. 28, 78–82. doi: 10.1016/j.yebeh.2013.04.003, PMID: 23666465

[ref34] Faingold C. L. Tupal S. Randall M. (2011). Prevention of seizure-induced sudden death in a chronic SUDEP model by semichronic administration of a selective serotonin reuptake inhibitor. Epilepsy Behav. 22, 186–190. doi: 10.1016/j.yebeh.2011.06.015, PMID: 21783426

[ref35] Fan H. C. Yang M.-T. Lin L.-C. Chiang K.-L. Chen C.-M. (2023). Clinical and genetic features of Dravet syndrome: a prime example of the role of precision medicine in genetic epilepsy. Int. J. Mol. Sci. 25:31. doi: 10.3390/ijms25010031, PMID: 38203200 PMC10779156

[ref36] Favero M. Sotuyo N. P. Lopez E. Kearney J. A. Goldberg E. M. (2018). A transient developmental window of fast-spiking interneuron dysfunction in a mouse model of Dravet syndrome. J. Neurosci. 38, 7912–7927. doi: 10.1523/JNEUROSCI.0193-18.2018, PMID: 30104343 PMC6125809

[ref37] Feng H. J. Faingold C. L. (2017). Abnormalities of serotonergic neurotransmission in animal models of SUDEP. Epilepsy Behav. 71, 174–180. doi: 10.1016/j.yebeh.2015.06.008, PMID: 26272185 PMC4749463

[ref38] Feng T. Makiello P. Dunwoody B. Steckler F. Symonds J. D. Zuberi S. M.. (2024). Long-term predictors of developmental outcome and disease burden in SCN1A-positive Dravet syndrome. Brain Commun. 6:fcae004. Published 2024 Jan 9. doi: 10.1093/braincomms/fcae004, PMID: 38229878 PMC10789590

[ref39] Feng Y. Shuman T. (2022). Blame it on the inputs: overexcited entorhinal inputs drive dentate gyrus hyperexcitability in a mouse model of Dravet syndrome. Epilepsy Curr. 22, 372–374. doi: 10.1177/15357597221112801, PMID: 36426186 PMC9661622

[ref40] Fine A. L. (2024). Envisioning a critical period to preserve development: communication delays in SCN1A+ dravet syndrome. Epilepsy Curr. 24, 342–344. doi: 10.1177/15357597241280687, PMID: 39508016 PMC11536425

[ref41] Fukushima K. Higashiyama H. Kazuta Y. Hashimoto K. Watanabe N. Furuya Y.. (2023). Discovery of E2730, a novel selective uncompetitive GAT1 inhibitor, as a candidate for anti-seizure medication. Epilepsia Open. 8, 834–845. doi: 10.1002/epi4.12741, PMID: 37052238 PMC10472371

[ref42] Gao M. M. Huang H. Y. Chen S. Y. Tang H. L. He N. Feng W. C.. (2021). The ALOXE3 gene variants from patients with Dravet syndrome decrease gene expression and enzyme activity. Brain Res. Bull. 170, 81–89. doi: 10.1016/j.brainresbull.2021.02.010, PMID: 33581311

[ref43] Gartside S. E. Cowen P. J. Sharp T. (1992). Effect of 5-hydroxy-L-tryptophan on the release of 5-HT in rat hypothalamus in vivo as measured by microdialysis. Neuropharmacology 31, 9–14. doi: 10.1016/0028-3908(92)90154-H, PMID: 1531865

[ref44] Giorgi S. Auvin S. Schoonjans A. S. Turón E. Sánchez-Miranda I. Gil-Nagel A.. (2024). A tool for Dravet syndrome-associated neuropsychiatric comorbidities evaluation (DANCE). Epilepsy Behav. 158:109958. doi: 10.1016/j.yebeh.2024.109958, PMID: 39067307

[ref45] Gonsales M. C. Montenegro M. A. Preto P. Guerreiro M. M. Coan A. C. Quast M. P.. (2019). Multimodal analysis of SCN1A missense variants improves interpretation of clinically relevant variants in Dravet syndrome. Front. Neurol. 10:289. doi: 10.3389/fneur.2019.00289, PMID: 31001185 PMC6455056

[ref46] Gorman K. M. Peters C. H. Lynch B. Jones L. Bassett D. S. King M. D.. (2021). Persistent sodium currents in SCN1A developmental and degenerative epileptic dyskinetic encephalopathy. Brain Commun. 3:fcab235. doi: 10.1093/braincomms/fcab235, PMID: 34755109 PMC8568850

[ref47] Griffin A. Hamling K. R. Knupp K. Hong S. G. Lee L. P. Baraban S. C. (2017). Clemizole and modulators of serotonin signalling suppress seizures in Dravet syndrome. Brain 140, aww342–aww683. doi: 10.1093/brain/aww342, PMID: 28073790 PMC6075536

[ref48] Griffin A. L. Jaishankar P. Grandjean J. M. Olson S. H. Renslo A. R. Baraban S. C. (2019). Zebrafish studies identify serotonin receptors mediating antiepileptic activity in Dravet syndrome. Brain Commun. 1:fcz008. doi: 10.1093/braincomms/fcz008, PMID: 31667472 PMC6798786

[ref49] Gu F. Parada I. Yang T. Longo F. M. Prince D. A. (2022). Chronic partial Trk B activation reduces seizures and mortality in a mouse model of Dravet syndrome. Proc. Natl. Acad. Sci. USA 119:e2022726119. doi: 10.1073/pnas.2022726119, PMID: 35165147 PMC8851461

[ref50] Guan L. Han Y. Yang C. Lu S. du J. Li H.. (2022). CRISPR-Cas9-mediated gene therapy in neurological disorders. Mol. Neurobiol. 59, 968–982. doi: 10.1007/s12035-021-02638-w, PMID: 34813019

[ref51] Haigh J. L. Adhikari A. Copping N. A. Stradleigh T. Wade A. A. Catta-Preta R.. (2021). Deletion of a non-canonical regulatory sequence causes loss of Scn1a expression and epileptic phenotypes in mice. Genome Med. 13:69. doi: 10.1186/s13073-021-00884-0, PMID: 33910599 PMC8080386

[ref52] Hameed M. Q. Hui B. Lin R. MacMullin P. C. Pascual-Leone A. Vermudez S. A. D.. (2023). Depressed glutamate transporter 1 expression in a mouse model of Dravet syndrome. Ann. Clin. Transl. Neurol. 10, 1695–1699. doi: 10.1002/acn3.51851, PMID: 37452008 PMC10502630

[ref53] Hammer M. F. Pan Y. Cumbay M. Pendziwiat M. Afawi Z. Goldberg-Stern H.. (2022). Whole exome sequencing and co-expression analysis identify an SCN1A variant that modifies pathogenicity in a family with genetic epilepsy and febrile seizures plus. Epilepsia 63, 1970–1980. doi: 10.1111/epi.17296, PMID: 35592948 PMC10753192

[ref54] Han Z. Chen C. Christiansen A. Ji S. Lin Q. Anumonwo C.. (2020). Antisense oligonucleotides increase Scn1a expression and reduce seizures and SUDEP incidence in a mouse model of Dravet syndrome. Sci. Transl. Med. 12:eaaz6100. doi: 10.1126/scitranslmed.aaz6100, PMID: 32848094

[ref55] Hanson J. E. Ma K. Elstrott J. Weber M. Saillet S. Khan A. S.. (2020). GluN2A NMDA receptor enhancement improves brain oscillations, synchrony, and cognitive functions in Dravet syndrome and Alzheimer's disease models. Cell Rep. 30, 381–396.e4. doi: 10.1016/j.celrep.2019.12.030, PMID: 31940483 PMC7017907

[ref56] Hatini P. G. Commons K. G. (2020). A 5-HT1D -receptor agonist protects Dravet syndrome mice from seizure and early death. Eur. J. Neurosci. 52, 4370–4374. doi: 10.1111/ejn.14776, PMID: 32394465 PMC7655614

[ref57] Hawkins N. A. Lewis M. Hammond R. S. Doherty J. J. Kearney J. A. (2017). The synthetic neuroactive steroid SGE-516 reduces seizure burden and improves survival in a Dravet syndrome mouse model. Sci. Rep. 7:15327. doi: 10.1038/s41598-017-15609-w, PMID: 29127345 PMC5681541

[ref58] Hawkins N. A. Nomura T. Duarte S. Barse L. Williams R. W. Homanics G. E.. (2021). Gabra2 is a genetic modifier of Dravet syndrome in mice. Mamm. Genome 32, 350–363. doi: 10.1007/s00335-021-09877-1, PMID: 34086081 PMC8458207

[ref59] Hawkins N. A. Zachwieja N. J. Miller A. R. Anderson L. L. Kearney J. A. (2016). Fine mapping of a Dravet syndrome modifier locus on mouse chromosome 5 and candidate gene analysis by RNA-seq. PLoS Genet. 12:e1006398. doi: 10.1371/journal.pgen.1006398, PMID: 27768696 PMC5074504

[ref60] Hernandez C. C. Tian X. J. Hu N. Shen W. Catron M. A. Yang Y.. (2021). Dravet syndrome-associated mutations in GABRA1, GABRB2 and GABRG2 define the genetic landscape of defects of *GABAA* receptors. Brain Commun. 3:fcab033. doi: 10.1093/braincomms/fcab033, PMID: 34095830 PMC8176149

[ref61] Hsiao J. Yuan T. Y. Tsai M. S. Lu C. Y. Lin Y. C. Lee M. L.. (2016). Upregulation of Haploinsufficient gene expression in the brain by targeting a Long non-coding RNA improves seizure phenotype in a model of Dravet syndrome. EBioMedicine 9, 257–277. doi: 10.1016/j.ebiom.2016.05.011, PMID: 27333023 PMC4972487

[ref62] Isom L. L. (2019). Is targeting of compensatory Ion Channel gene expression a viable therapeutic strategy for Dravet syndrome? Epilepsy Curr. 19, 193–195. doi: 10.1177/1535759719844780, PMID: 31035820 PMC6610383

[ref63] Jansen N. A. Dehghani A. Breukel C. Tolner E. A. van den Maagdenberg A. M. J. M. (2020). Focal and generalized seizure activity after local hippocampal or cortical ablation of NaV 1.1 channels in mice. Epilepsia 61, e30–e36. doi: 10.1111/epi.16482, PMID: 32190912 PMC7216883

[ref64] Khvorova A. Watts J. K. (2017). The chemical evolution of oligonucleotide therapies of clinical utility. Nat. Biotechnol. 35, 238–248. doi: 10.1038/nbt.3765, PMID: 28244990 PMC5517098

[ref65] Kim J. Hu C. Moufawad el Achkar C. Black L. E. Douville J. Larson A.. (2019). Patient-customized oligonucleotide therapy for a rare genetic disease. N. Engl. J. Med. 381, 1644–1652. doi: 10.1056/NEJMoa1813279, PMID: 31597037 PMC6961983

[ref66] Kong X. Dai G. Zeng Z. Zhang Y. Gu J. Ma T.. (2024). Integrating proteomics and transcriptomics reveals the potential pathways of hippocampal neuron apoptosis in Dravet syndrome model mice. Int. J. Mol. Sci. 25:4457. doi: 10.3390/ijms25084457, PMID: 38674042 PMC11050081

[ref67] Kress G. T. Chan F. Garcia C. A. Merrifield W. S. (2022). Utilizing machine learning algorithms to predict subject genetic mutation class from in silico models of neuronal networks. BMC Med. Inform. Decis. Mak. 22:290. doi: 10.1186/s12911-022-02038-7, PMID: 36352381 PMC9647930

[ref68] Kulikov A. V. Osipova D. V. Naumenko V. S. Popova N. K. (2005). Association between Tph2 gene polymorphism, brain tryptophan hydroxylase activity and aggressiveness in mouse strains. Genes Brain Behav. 4, 482–485. doi: 10.1111/j.1601-183X.2005.00145.x, PMID: 16268992

[ref69] Layer N. Sonnenberg L. Pardo González E. Benda J. Hedrich U. B. S. Lerche H.. (2021). Dravet variant SCN1AA1783V impairs interneuron firing predominantly by altered channel activation. Front. Cell. Neurosci. 15:754530 2021 Oct 28. doi: 10.3389/fncel.2021.754530, PMID: 34776868 PMC8581729

[ref70] Lenk G. M. Jafar-Nejad P. Hill S. F. Huffman L. D. Smolen C. E. Wagnon J. L.. (2020). Scn8a antisense oligonucleotide is protective in mouse models of SCN8A encephalopathy and Dravet syndrome. Ann. Neurol. 87, 339–346. doi: 10.1002/ana.25676, PMID: 31943325 PMC7064908

[ref71] Li W. Schneider A. L. Scheffer I. E. (2021). Defining Dravet syndrome: an essential pre-requisite for precision medicine trials. Epilepsia 62, 2205–2217. doi: 10.1111/epi.17015, PMID: 34338318 PMC9291974

[ref72] Li M. Yang L. Qian W. Ray S. Lu Z. Liu T.. (2023). A novel rat model of Dravet syndrome recapitulates clinical hallmarks. Neurobiol. Dis. 184:106193. doi: 10.1016/j.nbd.2023.106193, PMID: 37295561

[ref73] Mahdieh N. Mikaeeli S. Badv R. S. Shirazi A. G. Maleki M. Rabbani B. (2018). Pathogenic significance of SCN1A splicing variants causing Dravet syndrome: improving diagnosis with targeted sequencing for variants by in silico analysis. Clin. Neurol. Neurosurg. 166, 80–90. doi: 10.1016/j.clineuro.2018.01.030, PMID: 29408779

[ref74] Mantegazza M. Broccoli V. (2019). SCN1A/NaV 1.1 channelopathies: mechanisms in expression systems, animal models, and human iPSC models. Epilepsia 60, S25–S38. doi: 10.1111/epi.14700 31904127

[ref75] Martins Custodio H. Clayton L. M. Bellampalli R. Pagni S. Silvennoinen K. Caswell R.. (2023). Widespread genomic influences on phenotype in Dravet syndrome, a 'monogenic' condition. Brain 146, 3885–3897. doi: 10.1093/brain/awad111, PMID: 37006128 PMC10473570

[ref76] McKay R. A. Miraglia L. J. Cummins L. L. Owens S. R. Sasmor H. Dean N. M. (1999). Characterization of a potent and specific class of antisense oligonucleotide inhibitor of human protein kinase C-alpha expression. J. Biol. Chem. 274, 1715–1722. doi: 10.1074/jbc.274.3.1715, PMID: 9880552

[ref77] McNamara N. A. Dang L. T. Sturza J. Ziobro J. M. Fedak Romanowski E. M. Smith G. C.. (2020). Thrombocytopenia in pediatric patients on concurrent cannabidiol and valproic acid. Epilepsia 61, e85–e89. doi: 10.1111/epi.16596, PMID: 32614070

[ref78] Mich J. K. Ryu J. Wei A. D. Gore B. B. Guo R. Bard A. M.. (2025). Interneuron-specific dual-AAV SCN1A gene replacement corrects epileptic phenotypes in mouse models of Dravet syndrome. Sci. Transl. Med. 17:eadn5603. doi: 10.1126/scitranslmed.adn5603, PMID: 40106582

[ref79] Miljanovic N. van Dijk R. M. Buchecker V. Potschka H. (2021). Metabolomic signature of the Dravet syndrome: a genetic mouse model study. Epilepsia 62, 2000–2014. doi: 10.1111/epi.16976, PMID: 34223647

[ref80] Monia B. P. Lesnik E. A. Gonzalez C. Lima W. F. McGee D. Guinosso C. J.. (1993). Evaluation of 2′-modified oligonucleotides containing 2′-deoxy gaps as antisense inhibitors of gene expression. J. Biol. Chem. 268, 14514–14522. doi: 10.1016/S0021-9258(19)85268-7, PMID: 8390996

[ref81] Mora-Jimenez L. Valencia M. Sanchez-Carpintero R. Tønnesen J. Fadila S. Rubinstein M.. (2021). Transfer of *SCN1A* to the brain of adolescent mouse model of Dravet syndrome improves epileptic, motor, and behavioral manifestations. Mol. Ther. 25, 585–602. doi: 10.1016/j.omtn.2021.08.003, PMID: 34589280 PMC8463324

[ref82] Nakakubo S. Hiramatsu Y. Goto T. Kimura S. Narugami M. Nakajima M.. (2023). Therapeutic effects of KRM-II-81, positive allosteric modulator for α2/3 subunit containing GABAA receptors, in a mouse model of Dravet syndrome. Front. Pharmacol. 14:1273633. doi: 10.3389/fphar.2023.1273633, PMID: 37849734 PMC10577232

[ref83] Ng Y. T. Conry J. A. Drummond R. Stolle J. Weinberg M. A. OV-1012 Study Investigators (2011). Randomized, phase III study results of clobazam in Lennox-Gastaut syndrome. Neurology 77, 1473–1481. doi: 10.1212/WNL.0b013e318232de76, PMID: 21956725 PMC12477877

[ref84] Ngo T. T. D. Lea R. A. Maksemous N. Eccles D. A. Smith R. A. Dunn P. J.. (2021). The MinION as a cost-effective technology for diagnostic screening of the SCN1A gene in epilepsy patients. Epilepsy Res. 172:106593. doi: 10.1016/j.eplepsyres.2021.106593, PMID: 33721710

[ref85] Niibori Y. Lee S. J. Minassian B. A. Hampson D. R. (2020). Sexually divergent mortality and partial phenotypic rescue after gene therapy in a mouse model of Dravet syndrome. Hum. Gene Ther. 31, 339–351. doi: 10.1089/hum.2019.225, PMID: 31830809 PMC7087406

[ref86] Nordli D. R. 3rd Mclaren J. R. Araujo G. Gupta M. Galan F. (2024). Pediatric epilepsy syndromes with associated developmental impairment. Dev. Med. Child Neurol. 66, 691–701. doi: 10.1111/dmcn.15838, PMID: 38140949

[ref87] Ogiwara I. Miyamoto H. Morita N. Atapour N. Mazaki E. Inoue I.. (2007). Nav 1.1 localizes to axons of parvalbumin-positive inhibitory interneurons: a circuit basis for epileptic seizures in mice carrying an Scn1a gene mutation. J. Neurosci. 27, 5903–5914. doi: 10.1523/JNEUROSCI.5270-06.2007, PMID: 17537961 PMC6672241

[ref88] Osipova D. V. Kulikov A. V. Mekada K. Yoshiki A. Moshkin M. P. Kotenkova E. V.. (2010). Distribution of the C1473G polymorphism in tryptophan hydroxylase 2 gene in laboratory and wild mice. Genes Brain Behav. 9, 537–543. doi: 10.1111/j.1601-183X.2010.00586.x, PMID: 20398061

[ref89] Pan X. Li Z. Jin X. Zhao Y. Huang G. Huang X.. (2021). Comparative structural analysis of human Nav1.1 and Nav1.5 reveals mutational hotspots for sodium channelopathies. Proc. Natl. Acad. Sci. USA 118:e2100066118. doi: 10.1073/pnas.2100066118, PMID: 33712547 PMC7980448

[ref90] Patel R. V. Nanda P. Richardson R. M. (2024). Neurosurgical gene therapy for central nervous system diseases. Neurotherapeutics 21:e00434. doi: 10.1016/j.neurot.2024.e00434, PMID: 39191071 PMC11445594

[ref91] Perucca E. Bialer M. White H. S. (2023). New GABA-targeting therapies for the treatment of seizures and epilepsy: I. Role of GABA as a modulator of seizure activity and recently approved medications acting on the GABA system. CNS Drugs 37, 755–779. doi: 10.1007/s40263-023-01027-2, PMID: 37603262 PMC10501955

[ref92] Poliquin S. Nwosu G. Randhave K. Shen W. Flamm C. Kang J. Q. (2024). Modulating endoplasmic reticulum chaperones and mutant protein degradation in GABRG2 (Q390X) associated with genetic epilepsy with febrile seizures plus and Dravet syndrome. Int. J. Mol. Sci. 25:4601. doi: 10.3390/ijms25094601, PMID: 38731820 PMC11083348

[ref93] Ricci R. Colasante G. (2021). CRISPR/dCas9 as a therapeutic approach for neurodevelopmental disorders: innovations and limitations compared to traditional strategies. Dev. Neurosci. 43, 253–261. doi: 10.1159/000515845, PMID: 33940579

[ref94] Richards K. L. Milligan C. J. Richardson R. J. Jancovski N. Grunnet M. Jacobson L. H.. (2018). Selective NaV1.1 activation rescues Dravet syndrome mice from seizures and premature death. Proc. Natl. Acad. Sci. USA 115, E8077–E8085. doi: 10.1073/pnas.1804764115, PMID: 30076230 PMC6112713

[ref95] Ricobaraza A. Bunuales M. Gonzalez-Aparicio M. Fadila S. Rubinstein M. Vides-Urrestarazu I.. (2023). Preferential expression of SCN1A in GABAergic neurons improves survival and epileptic phenotype in a mouse model of Dravet syndrome. J. Mol. Med. (Berl) 101, 1587–1601. doi: 10.1007/s00109-023-02383-8, PMID: 37819378 PMC10697872

[ref96] Rubinstein M. Han S. Tai C. Westenbroek R. E. Hunker A. Scheuer T.. (2015). Dissecting the phenotypes of Dravet syndrome by gene deletion. Brain 138, 2219–2233. doi: 10.1093/brain/awv142, PMID: 26017580 PMC5022661

[ref97] Sala-Coromina J. Raspall-Chaure M. Marcé-Grau A. de la Ossa A. M. Macaya A. (2021). Early-onset eyelid stereotypies are a frequent and distinctive feature in Dravet syndrome. Seizure 92, 155–157. doi: 10.1016/j.seizure.2021.08.020, PMID: 34521063

[ref98] Salazar J. J. Satriano A. Matamoros J. A. Fernández-Albarral J. A. Salobrar-García E. López-Cuenca I.. (2023). Retinal tissue shows glial changes in a Dravet syndrome Knock-in mouse model. Int. J. Mol. Sci. 24:2727. doi: 10.3390/ijms24032727, PMID: 36769051 PMC9916888

[ref99] Schoonjans A. S. (2019). An old drug for a new indication: repurposing fenfluramine from an anorexigen to an antiepileptic drug. Clin. Pharmacol. Ther. 106, 929–932. doi: 10.1002/cpt.1469, PMID: 31116409

[ref100] Schoonjans A. (2021). Aggravation of valproic acid induced thrombocytopenia after the introduction of fenfluramine, a case report. Seizure 93, 60–62. doi: 10.1016/j.seizure.2021.09.012, PMID: 34706322

[ref101] Schuster J. Laan L. Klar J. Jin Z. Huss M. Korol S.. (2019). Transcriptomes of Dravet syndrome iPSC derived GABAergic cells reveal dysregulated pathways for chromatin remodeling and neurodevelopment. Neurobiol. Dis. 132:104583. doi: 10.1016/j.nbd.2019.104583, PMID: 31445158

[ref102] Shao E. Chang C. W. Li Z. Yu X. Ho K. Zhang M.. (2022). TAU ablation in excitatory neurons and postnatal TAU knockdown reduce epilepsy, SUDEP, and autism behaviors in a Dravet syndrome model. Sci. Transl. Med. 14:eabm5527. doi: 10.1126/scitranslmed.abm5527, PMID: 35476595 PMC9102397

[ref103] Shen W. de Hoyos C. L. Migawa M. T. Vickers T. A. Sun H. Low A.. (2019). Chemical modification of PS-ASO therapeutics reduces cellular protein-binding and improves the therapeutic index. Nat. Biotechnol. 37, 640–650. doi: 10.1038/s41587-019-0106-2, PMID: 31036929

[ref104] Shi X. He W. Guo S. Zhang B. Ren S. Liu K.. (2019). RNA-seq analysis of the SCN1A-KO model based on CRISPR/Cas9 genome editing technology. Neuroscience 398, 1–11. doi: 10.1016/j.neuroscience.2018.11.052, PMID: 30529264

[ref105] Silvennoinen K. Gawel K. Tsortouktzidis D. Pitsch J. Alhusaini S. van Loo K. M. J.. (2022). SCN1A overexpression, associated with a genomic region marked by a risk variant for a common epilepsy, raises seizure susceptibility. Acta Neuropathol. 144, 107–127. doi: 10.1007/s00401-022-02429-0, PMID: 35551471 PMC9217876

[ref106] Solaz S. Cardenal-Muñoz E. Muñoz A. Giorgi S. Pallardó F. V. Romá-Mateo C.. (2024). Navigating Dravet syndrome in Spain: a cross-sectional study of diagnosis, management, and care coordination. Epilepsia Open. 9, 1806–1815. doi: 10.1002/epi4.13012, PMID: 38984594 PMC11450586

[ref107] Soto Jansson J. Bjurulf B. Dellenmark Blom M. Hallböök T. Reilly C. (2024). Diagnosis, epilepsy treatment and supports for neurodevelopment in children with Dravet syndrome: caregiver reported experiences and needs. Epilepsy Behav. 151:109603. doi: 10.1016/j.yebeh.2023.109603, PMID: 38168600

[ref108] Sri Hari A. Banerji R. Liang L. P. Fulton R. E. Huynh C. Q. Fabisiak T.. (2023). Increasing glutathione levels by a novel posttranslational mechanism inhibits neuronal hyperexcitability. Redox Biol. 67:102895. doi: 10.1016/j.redox.2023.102895, PMID: 37769522 PMC10539966

[ref109] Stein R. E. Kaplan J. S. Li J. Catterall W. A. (2019). Hippocampal deletion of NaV1.1 channels in mice causes thermal seizures and cognitive deficit characteristic of Dravet syndrome. Proc. Natl. Acad. Sci. USA 116, 16571–16576. doi: 10.1073/pnas.1906833116, PMID: 31346088 PMC6697805

[ref110] Strzelczyk A. (2022). A practical guide to the treatment of Dravet syndrome with anti-seizure medication. CNS Drugs 36, 217–237. doi: 10.1007/s40263-022-00898-1, PMID: 35156171 PMC8927048

[ref111] Strzelczyk A. Lagae L. Wilmshurst J. M. Brunklaus A. Striano P. Rosenow F.. (2023). Dravet syndrome: a systematic literature review of the illness burden. Epilepsia Open. 8, 1256–1270. doi: 10.1002/epi4.12832, PMID: 37750463 PMC10690674

[ref112] Studtmann C. Ladislav M. Topolski M. A. Safari M. Swanger S. A. (2022). NaV1.1 haploinsufficiency impairs glutamatergic and GABAergic neuron function in the thalamus. Neurobiol. Dis. 167:105672. doi: 10.1016/j.nbd.2022.105672, PMID: 35219855 PMC8957548

[ref113] Sullivan J. Benítez A. Roth J. Andrews J. S. Shah D. Butcher E.. (2024). A systematic literature review on the global epidemiology of Dravet syndrome and Lennox-Gastaut syndrome: prevalence, incidence, diagnosis, and mortality. Epilepsia 65, 1240–1263. doi: 10.1111/epi.17866, PMID: 38252068

[ref114] Takado Y. Takuwa H. Sampei K. Urushihata T. Takahashi M. Shimojo M.. (2022). MRS-measured glutamate versus GABA reflects excitatory versus inhibitory neural activities in awake mice. J. Cereb. Blood Flow Metab. 42, 197–212. doi: 10.1177/0271678X211045449, PMID: 34515548 PMC8721779

[ref115] Tanenhaus A. Stowe T. Young A. McLaughlin J. Aeran R. Lin I. W.. (2022). Cell-selective adeno-associated virus-mediated SCN1A gene regulation therapy rescues mortality and seizure phenotypes in a Dravet syndrome mouse model and is well tolerated in nonhuman Primates. Hum. Gene Ther. 33, 579–597. doi: 10.1089/hum.2022.037, PMID: 35435735 PMC9242722

[ref116] Tang S. Stamberger H. Calhoun J. D. Weckhuysen S. Carvill G. L. (2025). Antisense oligonucleotides modulate aberrant inclusion of poison exons in SCN1A-related Dravet syndrome. JCI Insight 10:e188014. doi: 10.1172/jci.insight.188014, PMID: 39946203 PMC11981616

[ref117] Treiman D. M. (2001). GABAergic mechanisms in epilepsy. Epilepsia 42, 8–12. doi: 10.1046/j.1528-1157.2001.042suppl.3008.x 11520315

[ref118] Tsai C. W. Ho S. Y. Chen I. C. Chang K. C. Chen H. J. Tsai F. C.. (2025). Abnormal increased mTOR signaling regulates seizure threshold in Dravet syndrome. Neuropharmacology 262:110166. doi: 10.1016/j.neuropharm.2024.110166, PMID: 39374769

[ref119] Uchino K. Kawano H. Tanaka Y. Adaniya Y. Asahara A. Deshimaru M.. (2021). Inhibitory synaptic transmission is impaired at higher extracellular Ca2+ concentrations in Scn1a+/− mouse model of Dravet syndrome. Sci. Rep. 11:10634. doi: 10.1038/s41598-021-90224-4, PMID: 34017040 PMC8137694

[ref120] Uchino K. Tanaka Y. Ikezawa W. Deshimaru M. Kubota K. Watanabe T.. (2023). Astrocyte Ca2+ signaling is facilitated in Scn1a+/− mouse model of Dravet syndrome. Biochem. Biophys. Res. Commun. 643, 169–174. doi: 10.1016/j.bbrc.2022.12.084, PMID: 36610382

[ref121] Valassina N. Brusco S. Salamone A. Serra L. Luoni M. Giannelli S.. (2022). *Scn1a* gene reactivation after symptom onset rescues pathological phenotypes in a mouse model of Dravet syndrome. Nat. Commun. 13:161 2022 Jan 10. doi: 10.1038/s41467-021-27837-w, PMID: 35013317 PMC8748984

[ref122] Ventura R. Beltrán-Corbellini Á. Toledano R. Román I. S. M. García-Morales I. Gil-Nagel A. (2024). Epileptogenic focal lesions in Dravet syndrome: a warning to investigators. Epileptic Disord. 26, 173–180. doi: 10.1002/epd2.20191, PMID: 38116874

[ref123] Voskobiynyk Y. Battu G. Felker S. A. Cochran J. N. Newton M. P. Lambert L. J.. (2021). Aberrant regulation of a poison exon caused by a non-coding variant in a mouse model of Scn1a-associated epileptic encephalopathy. PLoS Genet. 17:e1009195. doi: 10.1371/journal.pgen.1009195, PMID: 33411788 PMC7790302

[ref124] Voskobiynyk Y. Paz J. T. (2024). Nav1.1gating against the current: Ndnf interneurons hold strong in Dravet syndrome. Epilepsy Curr. 24, 431–433. doi: 10.1177/15357597241284350, PMID: 39540129 PMC11556329

[ref125] Wallace A. Wirrell E. Kenney-Jung D. L. (2016). Pharmacotherapy for Dravet syndrome. Paediatr. Drugs 18, 197–208. doi: 10.1007/s40272-016-0171-7, PMID: 26966048

[ref126] Wengert E. R. Wagley P. K. Strohm S. M. Reza N. Wenker I. C. Gaykema R. P.. (2022). Targeted augmentation of nuclear gene output (TANGO) of Scn1a rescues parvalbumin interneuron excitability and reduces seizures in a mouse model of Dravet syndrome. Brain Res. 1775:147743. doi: 10.1016/j.brainres.2021.147743, PMID: 34843701

[ref127] Westenbroek R. Kaplan J. Viray K. Stella N. (2023). The serine hydrolase ABHD6 controls survival and thermally induced seizures in a mouse model of Dravet syndrome. Neurobiol. Dis. 180:106099. doi: 10.1016/j.nbd.2023.106099, PMID: 36990366 PMC10332637

[ref128] Weuring W. J. Singh S. Volkers L. Rook M. B. van ‘t Slot R. H. Bosma M.. (2020). NaV1.1 and NaV1.6 selective compounds reduce the behavior phenotype and epileptiform activity in a novel zebrafish model for Dravet syndrome. PLoS One 15:e0219106. Published 2020 Mar 5. doi: 10.1371/journal.pone.0219106, PMID: 32134913 PMC7058281

[ref129] Wong J. C. Shapiro L. Thelin J. T. Heaton E. C. Zaman R. U. D'Souza M. J.. (2021). Nanoparticle encapsulated oxytocin increases resistance to induced seizures and restores social behavior in Scn1a-derived epilepsy. Neurobiol. Dis. 147:105147. doi: 10.1016/j.nbd.2020.105147, PMID: 33189882 PMC7726060

[ref130] Xu X. Yang X. Wu Q. Liu A. Yang X. Ye A. Y.. (2015). Amplicon resequencing identified parental mosaicism for approximately 10% of "de novo" SCN1A mutations in children with Dravet syndrome. Hum. Mutat. 36, 861–872. doi: 10.1002/humu.22819, PMID: 26096185 PMC5034833

[ref131] Yamagata T. Raveau M. Kobayashi K. Miyamoto H. Tatsukawa T. Ogiwara I.. (2020). CRISPR/dCas9-based Scn1a gene activation in inhibitory neurons ameliorates epileptic and behavioral phenotypes of Dravet syndrome model mice. Neurobiol. Dis. 141:104954. doi: 10.1016/j.nbd.2020.104954, PMID: 32445790

[ref132] Yang C. Pan R. Y. Guan F. Yuan Z. (2024). Lactate metabolism in neurodegenerative diseases. Neural Regen. Res. 19, 69–74. doi: 10.4103/1673-5374.374142, PMID: 37488846 PMC10479854

[ref133] Yuan Y. Lopez-Santiago L. Denomme N. Chen C. O'Malley H. A. Hodges S. L.. (2024). Antisense oligonucleotides restore excitability, GABA signalling and sodium current density in a Dravet syndrome model. Brain 147, 1231–1246. doi: 10.1093/brain/awad349, PMID: 37812817 PMC10994531

[ref134] Zeng C. Long X. Cotten J. F. Forman S. A. Solt K. Faingold C. L.. (2015). Fluoxetine prevents respiratory arrest without enhancing ventilation in DBA/1 mice. Epilepsy Behav. 45, 1–7. doi: 10.1016/j.yebeh.2015.02.013, PMID: 25771493 PMC4424071

[ref135] Zhang X. Beaulieu J. M. Sotnikova T. D. Gainetdinov R. R. Caron M. G. (2004). Tryptophan hydroxylase-2 controls brain serotonin synthesis. Science 305:217. doi: 10.1126/science.1097540, PMID: 15247473

[ref136] Zhou X. Xu H. Cai X. Tang B. Liu X. Shi Y.. (2021). Differences in SCN1A intronic variants result in diverse aberrant splicing patterns and are related to the phenotypes of epilepsy with febrile seizures. Epilepsy Res. 176:106711. doi: 10.1016/j.eplepsyres.2021.106711, PMID: 34293681

[ref1] Zulfiqar Ali Q. Marques P. Selvarajah A. Tabarestani S. Sadoway T. Andrade D. M. (2020). Starting stiripentol in adults with dravet syndrome? Watch for ammonia and carnitine. Epilepsia 61, 2435–2441. doi: 10.1111/epi.16684, PMID: 33084037

